# Pathogen and drought stress affect cell wall and phytohormone signaling to shape host responses in a sorghum COMT *bmr12* mutant

**DOI:** 10.1186/s12870-021-03149-5

**Published:** 2021-08-21

**Authors:** Maya Khasin, Lois F. Bernhardson, Patrick M. O’Neill, Nathan A. Palmer, Erin D. Scully, Scott E. Sattler, Deanna L. Funnell-Harris

**Affiliations:** 1 Wheat, Sorghum and Forage Research Unit, USDA-ARS, 251 Filley Hall, University of Nebraska-East Campus, Lincoln, NE 68583 USA; 2 Department of Plant Pathology, University of Nebraska, Lincoln, NE 68583 USA; 3 Department of Agronomy and Horticulture, University of Nebraska, Lincoln, NE 68583 USA; 4 Stored Product Insect and Engineering Research Unit, Center for Grain and Animal Health, USDA-ARS, Manhattan, KS 66502 USA; 5 Department of Entomology, Kansas State University, Manhattan, KS 66502 USA

**Keywords:** Lignin, Monolignols, *bmr6*, *bmr12*, Drought, *Fusarium*, *Macrophomina*, Coexpression networks

## Abstract

**Background:**

As effects of global climate change intensify, the interaction of biotic and abiotic stresses increasingly threatens current agricultural practices. The secondary cell wall is a vanguard of resistance to these stresses. *Fusarium thapsinum* (Fusarium stalk rot) and *Macrophomina phaseolina* (charcoal rot) cause internal damage to the stalks of the drought tolerant C4 grass, sorghum (*Sorghum bicolor* (L.) Moench), resulting in reduced transpiration, reduced photosynthesis, and increased lodging, severely reducing yields. Drought can magnify these losses. Two null alleles in monolignol biosynthesis of sorghum (*brown midrib 6-ref*, *bmr6-ref*; cinnamyl alcohol dehydrogenase, CAD; and *bmr12-ref*; caffeic acid O-methyltransferase, COMT) were used to investigate the interaction of water limitation with *F. thapsinum* or *M. phaseolina* infection.

**Results:**

The *bmr12* plants inoculated with either of these pathogens had increased levels of salicylic acid (SA) and jasmonic acid (JA) across both watering conditions and significantly reduced lesion sizes under water limitation compared to adequate watering, which suggested that drought may prime induction of pathogen resistance. RNA-Seq analysis revealed coexpressed genes associated with pathogen infection. The defense response included phytohormone signal transduction pathways, primary and secondary cell wall biosynthetic genes, and genes encoding components of the spliceosome and proteasome.

**Conclusion:**

Alterations in the composition of the secondary cell wall affect immunity by influencing phenolic composition and phytohormone signaling, leading to the action of defense pathways. Some of these pathways appear to be activated or enhanced by drought. Secondary metabolite biosynthesis and modification in SA and JA signal transduction may be involved in priming a stronger defense response in water-limited *bmr12* plants.

**Supplementary Information:**

The online version contains supplementary material available at 10.1186/s12870-021-03149-5.

## Background

Field crops are often faced with numerous abiotic and biotic stressors and challenging environmental conditions, which will continue to intensify with the growing impacts of climate change. Sorghum [*Sorghum bicolor* (L.) Moench] is heat and drought tolerant, requires low nitrogen and water inputs, and is grown as a staple food crop, biofuel feedstock and animal feed worldwide [1–5]. Despite its resilience to many common stressors, several fungal stalk pathogens threaten its production. The purpose of this study was to assess whether lignin-altered lines were more susceptible or resistant to select fungal pathogens under well-watered and drought conditions.

*Fusarium thapsinum* Klittich, J.F. Leslie, P. E Nelson & Marasas 1997 (= *Gibberella thapsina* Klittich, J.F. Leslie, P. E Nelson & Marasas 1997) and *Macrophomina phaseolina* (Tassi) Goid. 1947 are fungal stalk pathogens of sorghum that can cause lodging and result in yield loss, especially under drought. These fungi are causal agents of Fusarium stalk rot and charcoal rot, respectively. Both species can grow endophytically (asymptomatically), and the latter has a very broad host range consisting of over 800 host plants [6]. Some isolates of *M. phaseolina* are viable at temperatures greater than 40 °C [7–10]. Both pathogens have been known as necrotrophic pathogens but may experience a biotrophic phase before switching to a necrotrophic phase [6, 11]. Stalk rots are the most damaging diseases to production with incidence up to 100% in some fields [12], which can lead to lodging and significant biomass losses due to harvesting difficulties [13].

**Fig. 1:**
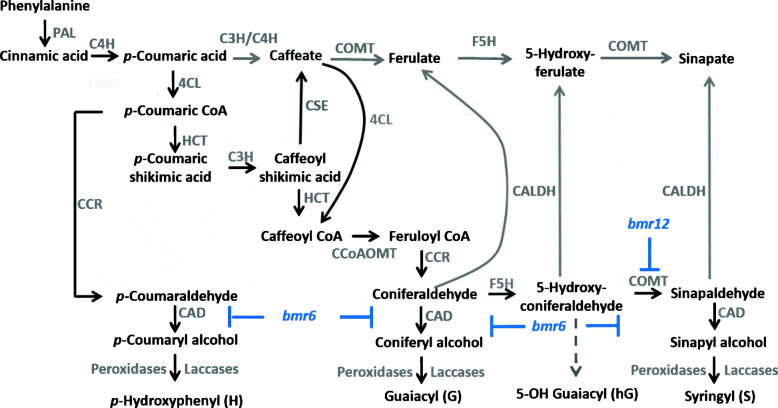
The phenylpropanoid biosynthesis pathway. The phenylpropanoid pathway produces phenolic compounds branching from phenylalanine ammonia lyase (PAL), including monolignols that lead to the biosynthesis of lignin subunits. The disruption of some of these enzymes results in *brown midrib* (*bmr*) mutants. Sorghum plants with *bmr12-ref* have a null mutation in caffeic acid O-methyltransferase (COMT) and plants with *bmr6-ref* have a null mutation in cinnamyl alcohol dehydrogenase (CAD). Both mutations result in altered lignification, such as the reduction of S-lignin in *bmr12*, and altered concentrations of wall-bound and soluble phenolics. Other abbreviations: C4H (cinnamate 4-hydroxylase), 4CL (4-coumarate: CoA ligase), HCT (p-hydroxycinnamoyltransferase), C3’H (4-coumarate hydroxylase), CSE (caffeoyl shikimate esterase), CCoAOMT (caffeoyl CoA-O-methyltransferase), CCR (cinnamoyl-CoA reductase), F5H (ferulate 5-hydroxylase)

Secondary cell wall lignification is a critical component of the plant stress response. Lignin provides mechanical support and a rigid, hydrophobic barrier against disease, herbivory, and abiotic stresses. Lignin is a complex and diversely cross-linked polymer whose biosynthesis proceeds through the phenylpropanoid pathway via phenylalanine ammonia lyase (PAL), linking primary metabolism to secondary metabolism [14, 15]. Phenylpropanoids are the precursors to lignin (Fig. 1). Intermediates in the phenylpropanoid pathway may feed into multiple metabolic pathways, initiating signal transduction networks that lead to defense responses and resistance to herbivores and pathogens [16–19]. It has been demonstrated that overexpression of *SbMyb60*, a transcription factor that controls monolignol biosynthesis, impacts phenolic content and secondary cell wall composition. Plants that overexpress *SbMyb60* have altered primary and secondary metabolism and defense pathways. These include leucine rich repeat-domain proteins (LRRs), cytochrome P450-domain proteins (Cyp450), redox-active proteins, and DNA replication and repair-associated proteins, highlighting the impact of the secondary cell wall on the defense response [14].

In cereal grasses, *brown midrib* mutants have a characteristic reddish-brown leaf midrib and are impaired in their ability to synthesize lignin. Eight loci that confer the brown midrib phenotype have been cloned and characterized in sorghum and maize and code for enzymes involved in monolignol biosynthesis [20–27]. In sorghum, *Brown midrib* (*Bmr*)*-12* and *Bmr6* encode two enzymes that catalyze the last two steps of monolignol biosynthesis, caffeic acid O-methyltransferase (COMT) and cinnamyl alcohol dehydrogenase (CAD), respectively (Fig. 1). Both *bmr6-ref* and *bmr12-ref* alleles contain nonsense mutations that result in complete loss of function [23, 28]. These mutants exhibit reduced levels of lignin and altered lignin composition within their cell walls relative to the wild-type [28, 29]. Impairing either of the last steps in monolignol biosynthesis was shown to lead to the accumulation of both soluble and cell wall bound hydroxycinnamic acids in *bmr6* and *bmr12* plants [28]. These mutants have facilitated the examination of the roles of these enzymes in disease responses.

Despite impaired lignification in *bmr* mutants, field studies have consistently demonstrated no increase in susceptibility of these mutants to natural disease occurrence and insect herbivory [30]. In some sorghum backgrounds, reduced susceptibility was observed in the *bmr6* and *bmr12* mutants [31, 32]. Mutants in *bmr6* have demonstrated increased resistance to the anthracnose disease, normally caused by the fungus *Colletotrichum sublineola* Henn. Ex Sacc. & Trotter 1904. The stalk pith from field-grown *bmr6* and *bmr12* plants was found to inhibit growth of laboratory-cultured fall armyworms ([*Spodoptera frugiperda* (J.E. Smith) (Lepidoptera: Noctuidae)]) and to a lesser extent corn earworms ([*Helicoverpa zea* (Boddie) (Lepidoptera: Noctuidae)], as compared with pith from wild-type plants, though this was affected by growth conditions of the stalks [30]. The *bmr* mutants across multiple genetic backgrounds had reduced incidences of field-grown grain infections by *Fusarium* and *Alternaria* species [32–34]. Accumulation of phenolics in *bmr6* and *bmr12* may be involved with the tolerance or even enhanced resistance to these fungal pathogens and to herbivory. At concentrations lower than the ones observed in the *bmr* mutants, these phenolic compounds limited the growth of some *Fusarium* species tested in vitro [35].

The current study measured lesion formation in Tx430 wild-type, *bmr6* and *bmr12* plants inoculated with *F. thapsinum* and *M. phaseolina* at 3 and 13 days after inoculation (DAI). It was found that *bmr12* plants showed reduced lesion length in response to fungal inoculation, but only under water limitation. This indicates that drought may drive a priming effect by activating generalized defense pathways in *bmr12* plants. This priming effect could be advantageous in halting disease progression. The *bmr12* plants had altered levels of soluble and wall-bound phenolics, as well as elevated levels of stress hormones jasmonic acid (JA) and salicylic acid (SA). Thus, phenolic metabolism altered by the *bmr12* mutation may lead to constitutive expression of some stress responses. In order to investigate this, gene coexpression network analysis was undertaken in tissues taken from plants immediately after inoculation (0 DAI), at lesion initiation (3 DAI), and at lesion expansion (13 DAI). We identified coexpression modules enriched for protein turnover, signal transduction, and primary and secondary metabolism that potentially contribute to the enhanced disease response in *bmr12*.

## Results

### *Responses of* bmr12 *and wild-type plants to inoculation with stalk pathogens* F. thapsinum *and* M. phaseolina

**Fig. 2:**
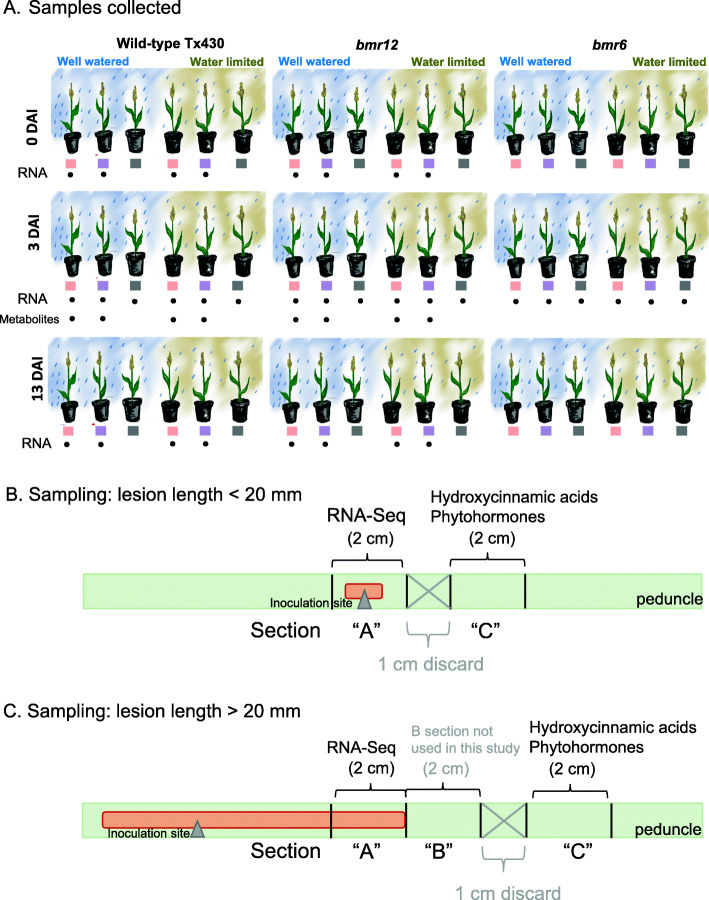
Experimental design of pathogen inoculation and watering treatments. A. The experimental design was a randomized split block by watering conditions with eight replicates over time and watered with a fertilizer-water mixture. RNA sequencing was performed on samples representing all water and inoculum treatments for all 3 days after inoculation (DAI), and performed on samples with PDB or *Fusarium thapsinum* and both water treatments for *bmr12* and the wild-type at 0 and 13 DAI, as indicated. Phytohormones and phenolic metabolites were analyzed from samples collected at 3 DAI from wild-type and *bmr12* plants with mock or *F. thapsinum* samples for both water treatments. B. Sample collection from plants with lesion lengths < 20 mm: A 2-cm section around the inoculation site was used for RNA sequencing (“A” section). Then, 1 cm distal to this section, a separate 2-cm tissue section was analyzed for phenolic metabolites and phytohormones (“C” section). C. Sample collection from plants with lesion lengths > 20 mm: A 2-cm section from the base of the lesion was used for RNA sequencing (“A” section). A 2-cm sample immediately adjacent to the base of the lesion (“B” section) was collected but not used in this study. Then, 1 cm distal to this section, a separate 2-cm tissue section was analyzed for phenolic metabolites and phytohormones (“C” section)

Well-watered and water limited Tx430 wild-type and near-isogenic *bmr6* and *bmr12* plants at anthesis were wound-inoculated at the peduncle with *F. thapsinum*, *M. phaseolina*, or potato dextrose broth (PDB) between anthesis and seed set. Water limitation was initiated at boot stage, where plants were only watered when soil moisture was below 25% field capacity as measured by a 10HS Moisture Sensor (Decagon Devices) probe with a U30 Shuttle (Hobo). Lesion formation, a plant response to wounding characterized by pigmentation along the length of the peduncle, was measured at 3 days after inoculation (DAI) (lesion initiation) and 13 DAI (lesion elongation). This assay is destructive; tissues sampled at 0, 3, and 13 DAI were measured on different plants inoculated at the same time (Fig. 2).

**Fig. 3:**
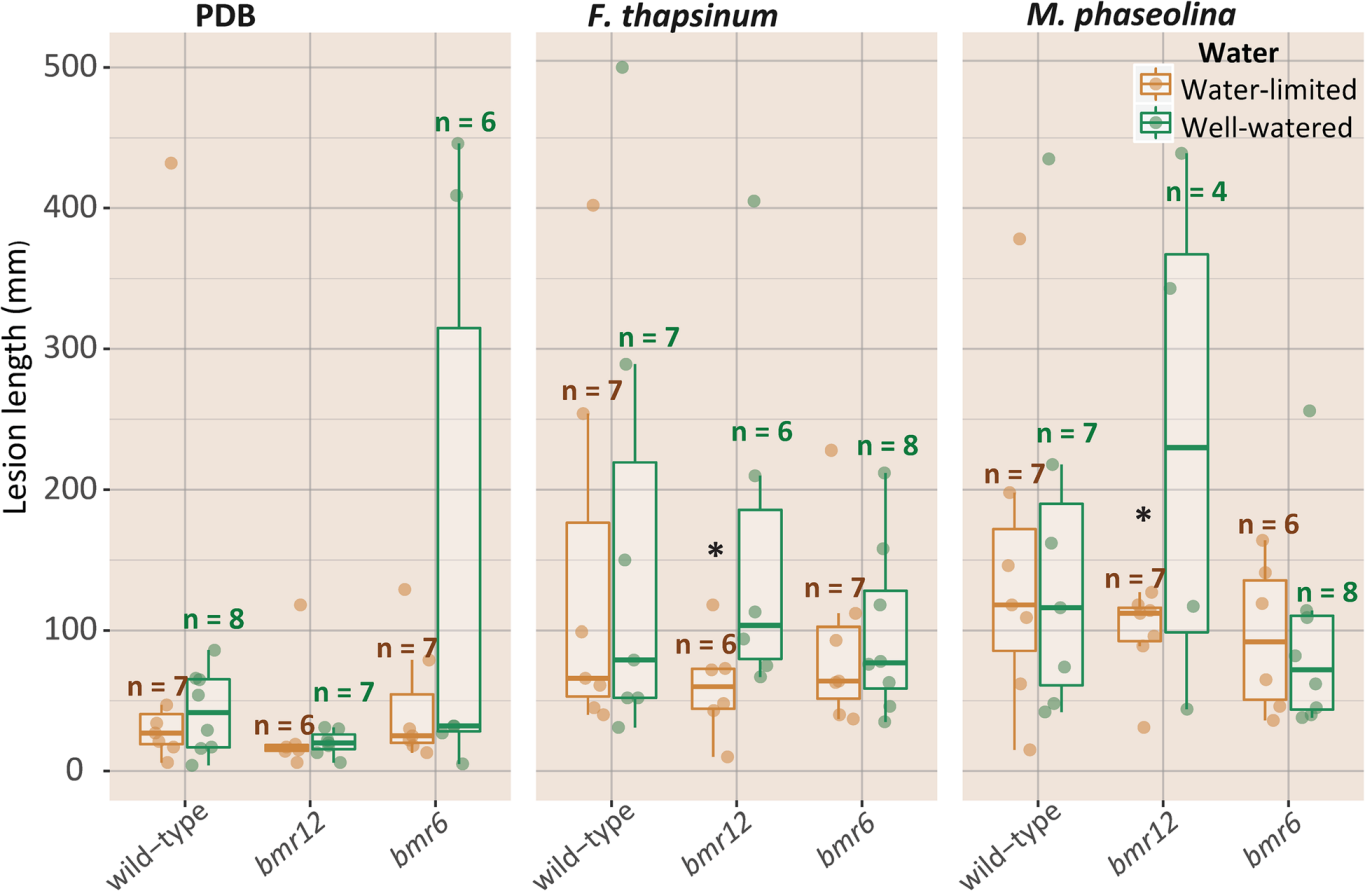
Reduced lesion sizes in *bmr12* under water limitation. Plants grown under well-watered and water-limited conditions were inoculated with mycelia-coated toothpicks with either *Fusarium thapsinum*, *Macrophomina phaseolina*, or PDB. Peduncles were harvested in a destructive assay at 0, 3, and 13 days after inoculation (DAI). At 13 DAI, *bmr12* plants displayed shorter lesions under water limitation in response to both stalk pathogens than under well-watered conditions. Asterisks denote significance at alpha = 0.05 using Fisher’s least squares means. Numbers above the boxes denote the number of plants (out of a maximum of eight) that were measured

A mixed linear model was fitted to the lesion length data (Table 1). The effects of plant genotype, inoculum treatment and DAI on lesion length were significant (*p* ≤ 0.04). The main effect of water treatment alone was not statistically significant (*p* = 0.16), but interactions with DAI (genotype: DAI) and the three-way interactions with DAI and plant genotype (water treatment: DAI:genotype) were significant (*p* ≤ 0.04). At 3 DAI, no significant differences were detected within the treatment groups (Fig. 3). At 13 DAI, there were significant differences in lesion lengths under the experimental conditions specified. When comparing *M. phaseolina*-inoculated plants under well-watered conditions, *bmr6* plants [Least Squares Means (LSM) ± Standard Error (SE): 93.3 ± 14.7] had significantly smaller mean lesions than wild-type (156.4 ± 26.6) (*p* = 0.04) (Table 1), while lesions produced on well-watered, *F. thapsinum*-inoculated *bmr6* (98.3 ± 13.3) were not significantly smaller than similarly-treated wild-type (156.0 ± 30.4) (*p* = 0.09). When considering the two water treatments with the same inoculum and plant genotype, *bmr12* plants under water-limitation conditions (*F. thapsinum*: 70.2 ± 20.0; *M. phaseolina*: 46.6 ± 24.3) had significantly shorter mean lesion lengths than when inoculated with respective pathogens under well-watered conditions (*F. thapsinum*: 161.2 ± 20.0; *M. phaseolina*: 197.4 ± 26.2) (*p* < 0.01) (Fig. 3). Because *bmr12* plants were more resistant to each pathogen than wild-type plants under water limitation, further analysis focused on this line. Also, *F. thapsinum*-inoculated plants were a focus because this pathogen is more commonly found on sorghum in Nebraska than *M. phaseolina*.

**Table 1:** ANOVA of fixed effects and interactions

Effect	Pr > F
Bmr	0.0425
Trt	<.0001
bmr*trt	0.2018
Water	0.1619
bmr*water	0.1993
trt*water	0.7013
bmr*trt*water	0.4399
Dai	<.0001
bmr*dai	0.0556
trt*dai	<.0001
bmr*trt*dai	0.4665
water*dai	0.0419
bmr*water*dai	0.0268
trt*water*dai	0.4972
bmr*trt*water*dai	0.3028

Plant genotype [*bmr*], inoculum treatment [trt], watering treatment [water] and days after inoculation [DAI], and interactions for inoculation of *brown midrib* (*bmr)*-*6 bmr12* and wild-type lines with two watering treatments and lesion measurement at 3 and 13 days after inoculation. The model assessed the interaction of watering condition × inoculum × timepoint × genotype and were analyzed for Levene’s homogeneity of variance and adjusted appropriately with replicate and replicate × water as random variables in the REPEATED/GROUP option

#### Measurement of phenolics and plant hormones at 3 DAI

**Fig. 4:**
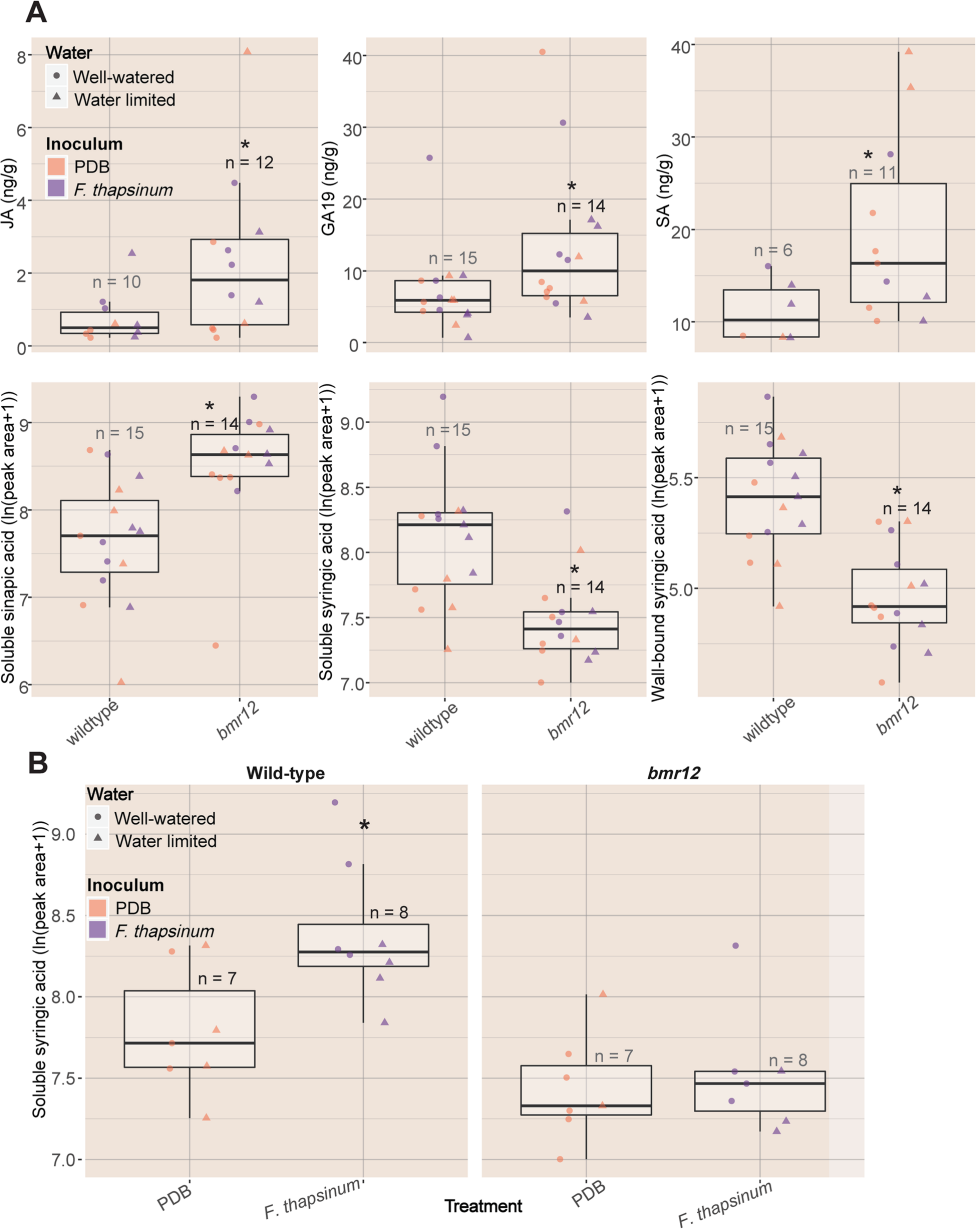
Levels of phytohormones and phenolic metabolites 3 days following inoculation with the stalk rot pathogen *F. thapsinum* or PDB inoculation. A. The *bmr12* plants contained elevated levels of GA19 (gibberellin A19, ng/g), JA (jasmonic acid, ng/g), SA (salicylic acid, ng/g), and elevated levels of sinapic acid (ln (peak area)) across all samples. Syringic acid (ln (peak area)) was elevated in wild-type plants inoculated with *F. thapsinum*, but not *bmr12*, which are deficient in S-lignin production. Pairwise p-values between *bmr12* and wild-type plants were calculated across all *bmr12* and wild-type plants, regardless of other conditions, by Wilcoxon rank-sum tests. They are presented separated by watering condition and inoculum. Numbers above the boxes denote the number of plants from which the specific compound was measured. Not all phytohormones were detected in all samples. Where phytohormones were not detected, the value of the limit of detection (LOD)/√2 was substituted and group means were compared by Wilcoxon rank-sum tests

Lesion formation necessitates the involvement of phenylpropanoid metabolism. At 3 DAI, hydroxycinnamic acids were measured in wild-type and *bmr12* tissues sampled at 3 DAI after inoculation with *F. thapsinum* and with PDB under both watering conditions (Fig. 2, Additional file 1). Due to limited tissue availability, there were not enough individual samples to meaningfully identify interacting effects of drought and disease, and comparisons in Fig. 4 are presented by genotype. Individual points are labeled with inoculum and watering condition (Fig. 4).

The *bmr12* plants had significantly elevated levels of sinapic acid, contrary to the model proposed in Fig. 1, which predicts that the synthesis of all sinapoyl groups would be impaired in *bmr12*. Wild-type plants infected with *F. thapsinum* had significantly higher concentrations of soluble syringic acid compared to PDB-inoculated controls; however, *bmr12* plants did not (Fig. 4B), as syringic acid production is impaired in *bmr12* plants (Fig. 1). The *bmr12* plants had decreased levels of wall-bound and soluble syringaldehyde, syringic acid, and 4-coumaric acid, as described previously [28] (Fig. 4).

Phytohormones initiate signal transduction cascades, including those active in defense. A broad spectrum of phytohormones were screened in tissues sampled at 3 DAI, the same tissues as were sampled for hydroxycinnamic acids. The *bmr12* plants also had significantly elevated levels of salicylic acid (SA), jasmonic acid (JA), gibberellin A19 (GA19), and significantly lower levels of wall-bound and soluble syringic acid (Fig. 4A). IAA-Aspartate (IAA-Asp) was detected only in a total of 12 plants, nine of which were inoculated with *F. thapsinum* and three of which were inoculated with PDB, however, the effect of inoculation with *F. thapsinum* was not significant as indicated by chi-square test (*p* = 0.16) (Additional file 1).

**Fig. 5:**
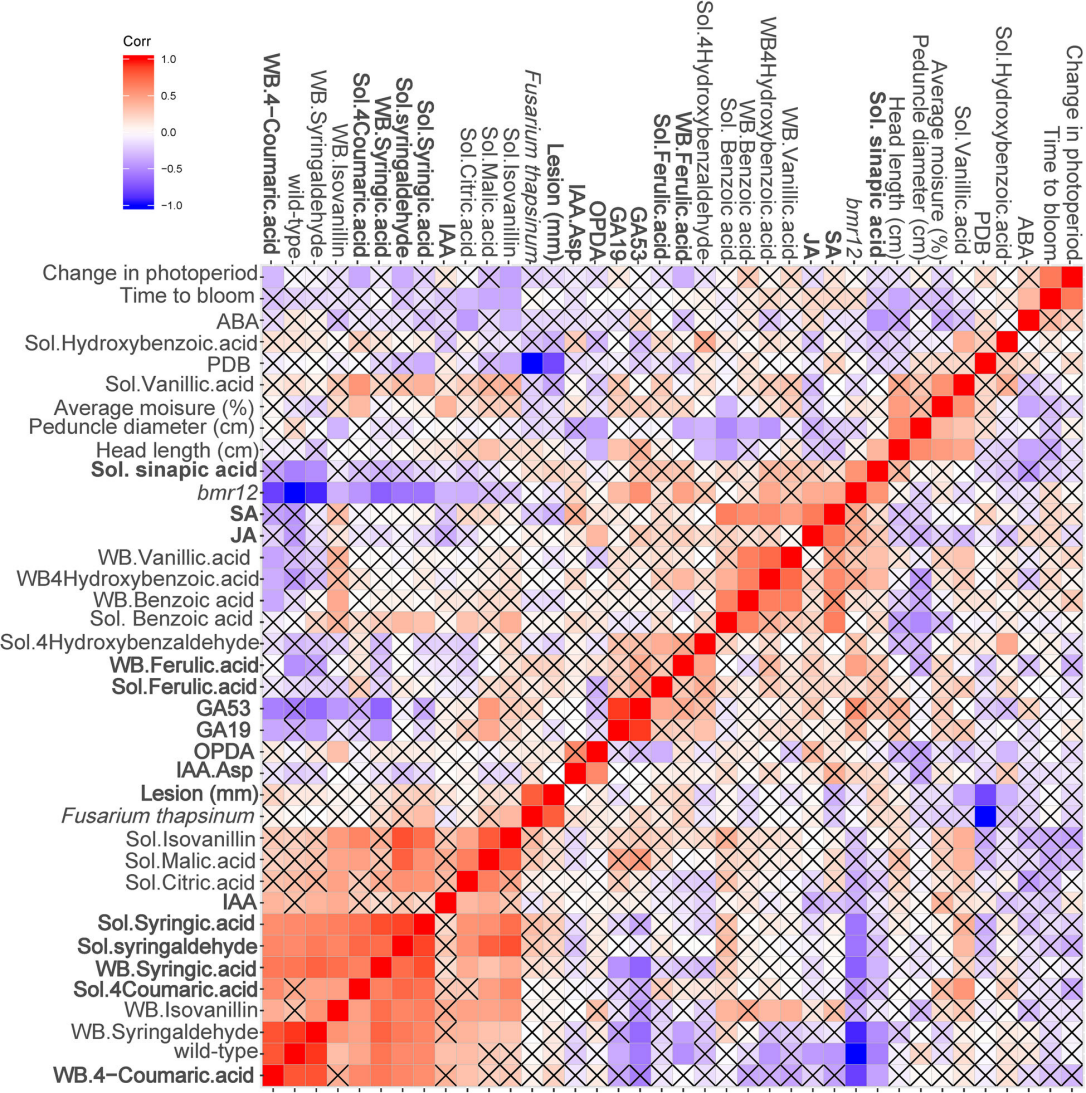
Correlation plot of physiological greenhouse characteristics and for phytohormones and phenolics. A Pearson correlation was calculated for recorded physiological characteristics for all plants sampled and for wall-bound and soluble phenolics (quantified by GC/MS) and phytohormones (quantified by LC/MS) assayed from a subset of samples. The R function hclust was used to hierarchically cluster these correlations. FDR-adjusted *p*-values (BH) are below the diagonal and comparisons with an X through them fail to meet the cutoff for alpha = 0.05

A correlation plot demonstrates phenolic and phytohormone trends associated with genotype, lesion length, and physiology (Fig. 5).

#### Network analysis highlights role of alternative splicing, protein turnover, and phenylpropanoids in disease response

Wild-type, *bmr6*, and *bmr12* plants inoculated with either fungus or PDB were sampled at 3 DAI to identify candidate early genes responsible for increased resistance*.* The study was then expanded to include 0 and 13 DAI for wild-type and *bmr12* with *F. thapsinum* and PDB inoculations under both watering treatments as described above (Fig. 2). Coexpression analysis was then performed with WGCNA. Genes were assigned to coexpression modules, and the correlation of the first principle component of the module with physiological traits (and metabolite levels, at 3 DAI) measured on those same plants was calculated. Modules were arbitrarily assigned color names by WGCNA. The qPCR validation of phenylpropanoid genes confirmed the expression pattern of the RNA-Seq data (additional file 15).

**Fig. 6:**
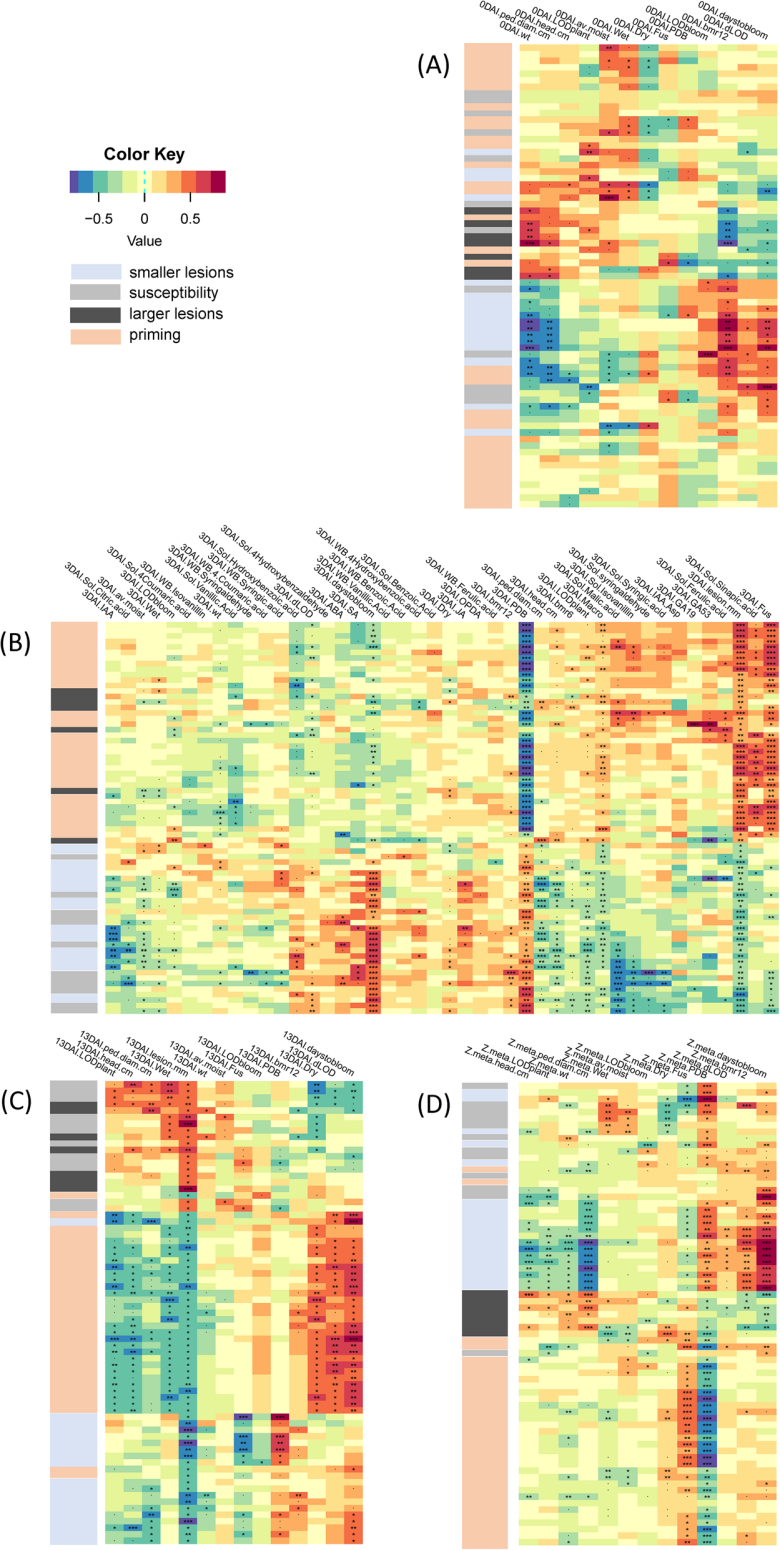
Gene-trait correlation for the lesion subset calculated by weighted correlation network analysis (WGCNA). Rows and columns were both hierarchically clustered A) at 0 DAI, B) at 3 DAI, C) at 13 DAI, and D) assigned a Z-score across all days. Each row represents a gene and they are coded as “smaller lesion”, “susceptibility”, “larger lesions”, and “priming” and as labeled in the key (Table 2). The correlation of genes within the wound subset to traits at 3 days after inoculation (DAI, above) and 13 DAI (below), including correlations to greenhouse data at both days sampled and to phytohormone and phenolic content at 3 DAI. Complete data are available in Additional file 2. Significance codes: . = *p* < 0.1; * = *p* < 0.05, ** = p < 0.01, *** = *p* < 0.001)

Gene expression correlations to physiological traits and metabolite measurements from the same plants sampled for RNA-Seq were calculated using Pearson correlation in WGCNA. These correlations were then hierarchically clustered along rows (genes) and columns (traits) to identify patterns in these correlations in tissues sampled from all days (Fig. 6). Genes were identified by their correlation to lesion length, as summarized in Table 2. Priming genes are of interest because of the potential drought priming effect in *bmr12.* Genes strongly positively correlated with both large and small lesions are also of interest, especially if they are agnostic to inoculum.

**Table 2:** Genes categorized based on the correlation of their expression with lesion length (α = 0.05)

Lesion category	Significant at 3 DAI	Significant at 13 DAI
Constitutive lesion	+	+
Early response	+	NS
Late response	NS	+
Priming	+	–
Susceptibility	–	+
Shorter lesion	–	–

Genes were categorized by their correlation with lesion length. Constitutive lesion genes were those whose expression was positively correlated with lesion length on both 3 and 13 days after inoculation (DAI), and smaller lesions displayed negative correlation with lesion length at both days. Putative priming genes were positively correlated with lesion length at 3 DAI but negatively correlated at 13 DAI. Putative susceptibility genes were negatively correlated with lesion length at 3 DAI, but positively correlated with lesion length at 13 DAI. Symbols: positive correlation (+), negative correlation (−), no significant correlation (NS)

**Fig. 7:**
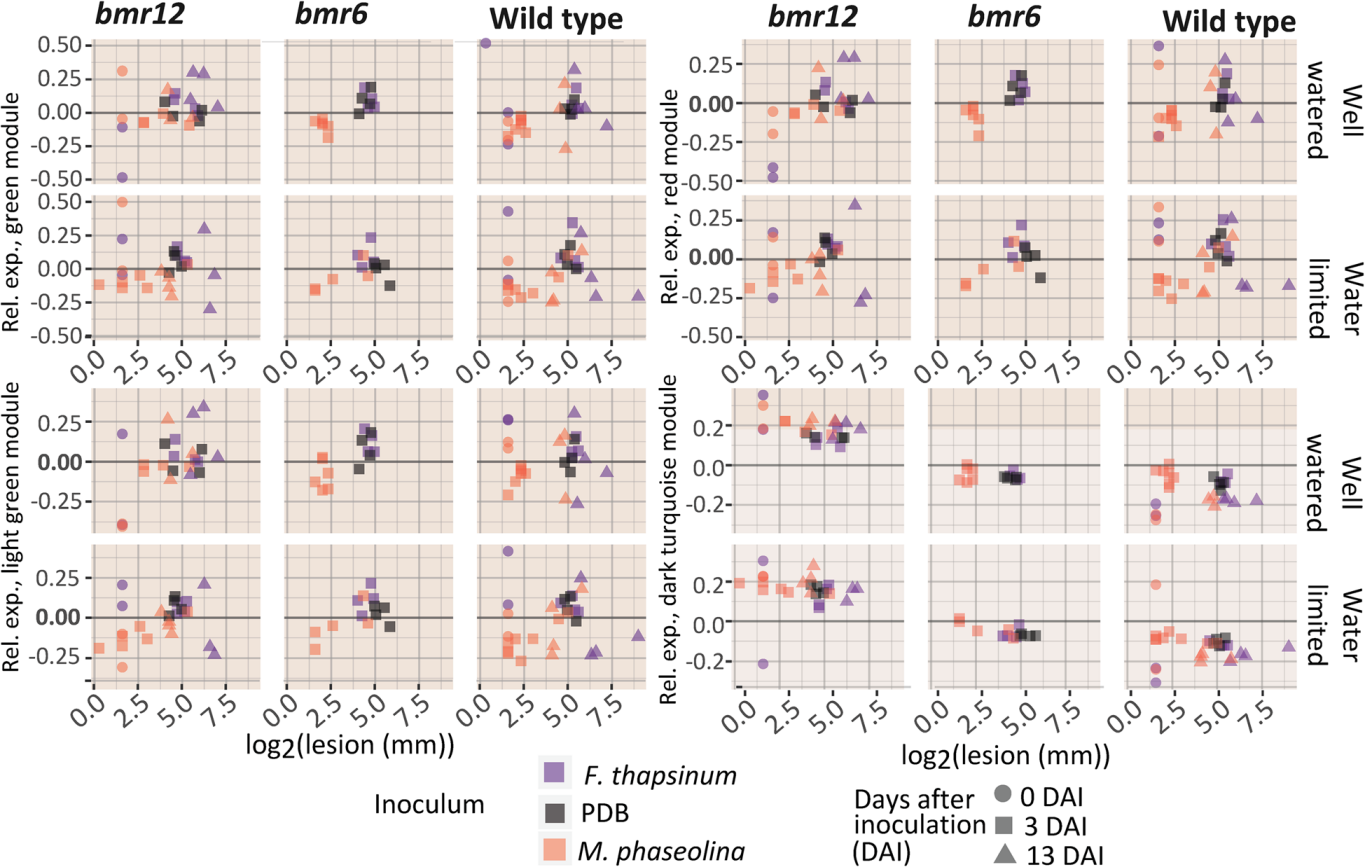
Relative expression of key module eigengenes. Coexpression modules were calculated by weighted correlation network analysis (WGCNA) and assigned color labels for referential convenience. The module eigengene refers to the first principle component (or the dominant expression profile) of a module. The green, light green, and red modules contained lesion response genes, and the dark turquoise module was constitutively associated with *bmr12* across all other conditions. The x-axis represents the log2 of the lesion length

Genes from the lesion-related subset (Table 2) were represented in three key modules: green, red, and light green, containing genes associated with defense responses (Fig. 7). Complete module eigengene expression data can be found in Additional file 5. In Fig. 7, each point represents the relative expression of a module in a sample plotted against log_2_-transformed lesion lengths. The relative expression of the dark turquoise module is considerably higher in *bmr12* samples regardless of lesion length.

Putative priming and smaller lesion-associated genes are closely associated with the *bmr12* genotype in tissues sampled at day 0 (Fig. 6A, Additional files 3 and 4). At 3 DAI, priming genes cluster with sinapic acid and *F. thapsinum* inoculation, although elevated sinapic acid in response to *F. thapsinum* inoculation was not detected. This could mean that they activate similar pathways. Sinapic acid levels were elevated in *bmr12* in tissues sampled at 3 DAI (Fig. 4A). At 3 DAI, smaller lesion and susceptibility genes also tend to cluster with days to bloom, water limitation treatment, and *bmr12* (Fig. 6B)*.* Finally, priming genes cluster together with water limitation, time to bloom, and change in photoperiod length at 13 DAI (Fig. 6C). Priming genes were associated with *F. thapsinum* infection and the water limitation treatment. In a meta-analysis conducted across all 3 days, shorter lesion-associated genes were positively correlated with *bmr12*, PDB inoculation, time to bloom, and change in photoperiod length.

#### Coexpression modules at 3 DAI associated with inoculum and time to bloom

##### Enrichment for photosynthesis and plant hormone signal transduction in modules negatively correlated with lesion length at 3 DAI

The turquoise module was negatively correlated with lesion length and positively correlated with PDB inoculation (Additional file 3). It was enriched for photosynthesis (photosystems I and II, antenna proteins, chlorophyll biosynthesis, and carbon fixation), primary metabolism, ribosomal proteins, and peroxisome (Table 3, Additional file 4). Constitutively smaller lesion genes were also positively correlated with time to bloom, *bmr12*, and PDB (Additional file 2, Fig. 6C).

**Table 3:** Modules obtained following weighted correlation network analysis (WGCNA), their gene contents, and selected KEGG enrichment

Module Label	Number of genes	Selected KEGG enrichment
Turquoise	1105	Photosynthesis, central metabolism, starch and sucrose metabolism, peroxisome
Blue	842	Ubiquitin mediated proteolysis, Protein processing in endoplasmic reticulum, One carbon pool by folate, mRNA surveillance pathway, Histidine metabolism, RNA transport
Brown	666	Spliceosome, ubiquitin mediated proteolysis, proteasome, RNA transport, base excision repair
Yellow	665	Spliceosome, RNA transport
Green	594	Plant-pathogen interaction (ETI)
Red	550	Primary metabolism, proteasome, phagosome, ER protein processing, alpha-linolenic acid metabolism, phenylpropanoid biosynthesis
Black	545	Plant hormone signal transduction (primarily JA), aminoacyl-tRNA biosynthesis
Pink	539	Plant hormone signal transduction (primarily IAA and ABA), ubiquitin mediated proteolysis, mRNA surveillance pathway
Magenta	536	Ribosome, central metabolism, amino acid biosynthesis, RNA transport
Purple	511	Primary metabolism, phagosome
Green-yellow	454	Starch and sucrose metabolism
Tan	454	Spliceosome, peroxisome
Salmon	450	Phenylpropanoid metabolism
Cyan	405	Endocytosis
Midnight blue	381	Phenylpropanoid biosynthesis, glutathione metabolism, glycosaminogen degradation, ubiquinone and other terpenoid-quinone biosynthesis
Light cyan	358	Significant annotated KEGG enrichment not detected
Grey60	348	Ribosomal proteins, primary metabolism, amino acid biosynthesis, RNA polymerase
Light green	344	Flavonoid biosynthesis [six chalcone isomerase paralogs], proteasome, fatty acid degradation, glutathione metabolism, peroxisome, protein processing in the ER
Light yellow	319	Plant hormone signal transduction (ARF, DELLA, COI, MPK6)
Royal blue	251	Circadian rhythm, thiamine metabolism, protein export
Dark red	249	Significant annotated KEGG enrichment not detected
Dark green	245	Gly/Ser/Thr metabolism
Dark turquoise	232	Significant annotated KEGG enrichment not detected
Dark grey	230	Selenocompound metabolism, ribosome, phosphatidylinositol signaling
Orange	217	N-glycan metabolism, sphingolipid metabolism, unfolded protein response-related proteins
Dark orange	207	N glycan biosynthesis, sphingolipid metabolism, purine metabolism, protein processing in the ER
White	202	primary metabolism, propanoate metabolism, beta-alanine metabolism, fatty acid degradation
Sky blue	201	Significant annotated KEGG enrichment not detected
Saddle brown	197	protein processing in the ER/plant pathogen interaction (same set of genes, Hsp70 family)
Steel blue	174	Ribosome biogenesis
Pale turquoise	170	Spliceosome, RNA transport, and basal transcription factors

Modules calculated by WGCNA revealed coexpression patterns enriched for annotated KEGG pathways

The tan and black module eigengenes were positively correlated with PDB and were negatively correlated with lesion length. The tan module was enriched for the spliceosome, including spliceosomal proteins and the exon-junction complex. The black module was enriched for genes related to plant hormone signal transduction, including genes encoding several of the core JA signaling components, COI, JAZ, JAR1, and MYC, the ABA signal transducer PP2C30, and the IAA signaling components TIR1, IAA15, and GH3.8 (Additional file 4). A gene encoding malate dehydrogenase in this module (Sobic.001G073900) was positively correlated with time to bloom and *bmr12*, and negatively correlated with lesion length, IAA and GA53 content. The expression level of this malate dehydrogenase was not influenced by inoculum.

The green-yellow module eigengene was positively correlated with PDB inoculation and time to bloom. This module was enriched for starch and sucrose metabolism and amino sugar and nucleotide sugar metabolism. This module contained predicted susceptibility genes, including a CASP-like protein 2A1 ortholog (Sobic.006G037300) and a stress-response A/B barrel domain-containing protein ortholog (Sobic.008G035400).

The salmon module was positively correlated with PDB inoculation and time to bloom. The salmon module was enriched for phenylpropanoid biosynthesis, including four peroxidases, PAL, CCoAOMT, and CCR1 (Additional file 3). *SbCAD5*, a cinnamyl alcohol dehydrogenase (Sobic.007G076000), is a candidate susceptibility gene in this module.

The light cyan and dark turquoise modules were correlated with *bmr12* (at 0 DAI). No significant KEGG enrichment was detected in these modules. The light cyan module eigengene was also positively correlated with ABA and SA content, shorter lesions, and time to bloom; the dark turquoise module was also positively correlated with the phenolic and phytohormone profile of *bmr12* (Fig. 7, Additional file 3).

##### Primary metabolism, protein turnover, and calcium-mediated signal transduction were enriched in modules associated with fungal inoculation at 3 DAI

The magenta module was positively correlated with *F. thapsinum* and *M. phaseolina* inoculation, lesion length, GA53, and soluble malic acid. A putative sulfoquinovosyl transferase (Sobic.002G000600) is a priming gene positively correlated with soluble sinapic acid levels and negatively correlated with abscisic acid content.

The white module was enriched for carbon metabolism. It contained genes coding for glutathione S-transferase (GST) (Sobic.001G318900), a GDSL esterase/lipase (Sobic.003G018800), and a catalase (Sobic.004G0115660) that were putative priming genes, indicative of a response to oxidative stress. The GST and GDSL esterase/lipase were positively correlated with sinapic acid content. The white module also contained an early response LRR-like gene (Sobic.010G217400) and *SbTCP5* (Sobic.002G198400), a transcription factor strongly positively correlated with water limitation.

The light green module was enriched for flavonoid biosynthesis (chalcone synthases 1, 2, 4, 6, and 7), protein processing in the ER, proteasome, and peroxisome. Several of the genes coding for flavonoid-related enzymes, including chalcone synthases 4 (Sobic.005G136300) and 6 (Sobic.005G137300/), a eugenol O-methyltransferase-like (Sobic.007G058800), and a putative flavonone 2-hydroxylase (Sobic.002G000400), were identified as putative priming genes. An early response tricin synthase (Sobic.007G218700) was strongly correlated with water limitation. The light green module also contained an RGA3-like disease resistance protein (Sobic.007G058800), also a putative priming gene. Early fungal response genes in this module included pathogenesis-related thaumatin-like protein (Sobic.001G145700) and chalcone synthase I (Sobic.005G137000). Additionally, three predicted chitinases (Sobic.005G084300, Sobic.006G132400, and Sobic.009G130100) and four pathogenesis-related (PR) proteins (Sobic.001G145700, Sobic.002G023300, Sobic.005G169200, and Sobic.005G169400) were early response genes in this module, highlighting the plant-pathogen interaction at the cell wall. These chitinases and pathogenesis-response proteins were associated with *Fusarium* infection across the three timepoints, and chitinases Sobic.009G130100 and Sobic.006G132400 were associated with the water limitation treatment across the three timepoints.

The green module is enriched for plant pathogen interactions through calcium-dependent protein kinases and calmodulin-dependent signal transduction cascades (Additional file 3). This module also contained putative priming genes, including a receptor kinase-like gene (Sobic.002G195800) that was positively correlated with soluble sinapic acid and soluble ferulic acid content, and negatively correlated with soluble hydroxybenzoic acid content. An abscisic stress-ripening protein 3-like gene (Sobic.006G078400) was also identified as a putative priming gene in this module. Early fungal response genes in the green module also included a LRR protein (Sobic.005G060900), a peroxidase (Sobic.009G033300), and an endochitinase (Sobic.006G132500), as well as plant hormone signal transduction genes such as an NPR1 ortholog (Sobic.003G032000), an AP2/ERF transcription factor, *SbEREB93* (Sobic.006G168100), a PP2C-like gene (Sobic.001G462800), and a calcium-dependent protein kinase (Sobic.004G279100).

The dark orange module contained an anthocyanidin 5,3-O-glucosyltransferase (Sobic.003G287600), and a flavonoid 3′-monooxygenase (Sobic.004G200800) as putative priming genes.

##### Oxylipin metabolism, pathogen response factors, protein turnover and ribosomal proteins were enriched in modules associated with F. thapsinum at 3 DAI

The red, orange, steel blue, and grey60 modules were associated with *F. thapsinum* inoculation. The red module was enriched for central metabolism, alpha-linolenic acid metabolism (involved in oxylipin biosynthesis, including three OPDA reductases, the committing step of OPDA biosynthesis), phagosome, protein processing in the endoplasmic reticulum, phenylpropanoid biosynthesis, proteasome subunits, cysteine and methionine metabolism (including ACC oxidase, the first committed step of ethylene biosynthesis), and the citric acid cycle. It contained a gene coding for an acetylserotonin methyltransferase (Sobic.005G216100), which was positively correlated with water limitation and was an early response gene. The red module also contained priming genes, including a dirigent protein-like ortholog (Sobic.005G101600), which was also correlated with water limitation. This module also contained two sets of PR proteins that were directly adjacent to one another on the same chromosome (Sobic.001G401100 and Sobic.001G401200; and Sobic.001G400700, Sobic.001G400800, and Sobic.001G400900). Sobic.001G400800 and Sobic.001G400900 were putative priming genes and Sobic.001G401100 and Sobic.001G401200 were early response genes. Another early response gene included in this module was a laccase (Sobic.005G215300) that was correlated with *bmr12* across the three timepoints. Four LRR-like genes (Sobic.004G124200, Sobic.005G126200, Sobic.010G061300, and Sobic.010G191750) were found in this module.

The orange module was enriched for ribosomal proteins and RNA transport (translation initiation factors and components of the nuclear pore complex) and contained a putative secreted peroxidase (Sobic.007G014200) that was identified as a larger lesion gene. The steel blue module was enriched for ribosomal proteins (components of the large and small subunits) and ribosome biogenesis, including small nucleolar ribonucleoproteins (Additional file 3). It contained an early response gene encoding a PR protein (Sobic.002G105300). The grey60 module was enriched with ribosomal proteins, glutathione metabolism, and glycolysis (Additional file 3).

##### RNA transport, spliceosome, and protein turnover pathways were enriched in modules associated with M. phaseolina at 3 DAI

There were fewer modules associated with only *M. phaseolina* inoculation compared to module eigengenes associated with *F. thapsinum* or with both fungi. *M. phaseolina* inoculation was correlated with the brown, dark green and cyan module eigengenes. The brown module was enriched for RNA transport (eukaryotic translation initiation factors; eIFs), ubiquitin mediated proteolysis (E3 ubiquitin ligases), proteasome (proteasome subunits), and spliceosome (DEAD-box RNA helicases, splicing factors). It contained five out of the seven constitutive lesion genes including an LG2 transcription-factor like gene (Sobic.003G363600). This module also included a peroxidase 47-like protein (Sobic.007G014200), which was not influenced by genotype but was positively correlated with soluble ferulic acid and GA53. Isoflavone reductase (Sobic.003G104350) was identified as an early response gene in this module. *SbCAD4* (Sobic.002G195600) was also correlated with the *bmr6* mutant, corroborating previous research identifying this gene as upregulated in *bmr6* plants compared to wild-type [23, 36]. Enrichment was not detected in the dark green nor the cyan module eigengenes.

##### Modules associated with other physiological characteristics at 3 DAI

The saddle brown module eigengene was positively correlated with the well-watered treatment. It was enriched for protein processing in the endoplasmic reticulum, plant-pathogen interactions (primarily through heat shock proteins) and the spliceosome. This was the only module correlated to watering treatment.

The midnight blue module eigengene was positively correlated with time to bloom. It was enriched for DNA replication (the MCM helicase complex), phenylpropanoid biosynthesis, and glutathione metabolism. It also contained a putative priming gene, a chalcone synthase 5 (Sobic.005G136200). The midnight blue module also contained the transcription factors *SbGLK7* (Sobic.002G016300), which was strongly positively correlated with water limitation and *bmr12* and negatively correlated with soluble malic acid, and *SbMyb60* (Sobic.004G273800), which impacts phenylpropanoid biosynthesis and lignin formation [14, 37]. *SbCAD5* (Sobic.004G071000) was strongly positively correlated with the *bmr6-ref* in this module, as described earlier.

The purple module was positively correlated with time to bloom and with JA and SA content. Its enrichment included oxidative phosphorylation (V-type ATPase subunits A, a, c, C, D, e, F; F-type ATP delta, epsilon; cyt b-c1 oxidase), carbon metabolism, TCA cycle, pyruvate metabolism, and phagosome (26 s proteasome subunits) (Additional file 4). The blue module was positively correlated with IAA-Asp, GA53, and ferulic acid content. This was enriched for ubiquitin-mediated proteolysis (ubiquitin ligase complex; sumo and ubiquitin conjugating enzymes), protein processing in the ER (ER-associated degradation complex), one carbon pool by folate (dihydrofolate reductase), and mRNA surveillance pathways (cleavage factors; EJC, PP2A) (Additional file 4).

#### Coexpression modules at 13 DAI correspond to physiological traits and link drought and disease tolerance

The turquoise module was correlated with PDB and with smaller lesions and was the only module correlated with inoculum at 13 DAI. The light cyan, purple, and dark turquoise modules were also positively correlated with time to bloom. The blue, yellow, brown, and pale turquoise modules were correlated with the wild-type. The pink, dark red, and green-yellow modules were negatively correlated with time to bloom.

Most priming genes at 13 DAI were positively correlated with water limitation treatment, change in photoperiod length, and time to bloom. In tissues sampled at 13 DAI (lesion expansion), correlations to physiological traits unrelated to inoculum dominated the module-trait relationships. In tissues sampled at day 13, these physiological processes were variously represented by the sky blue, dark orange, green, royal blue, light green, white, midnight blue, and red modules and were positively correlated with time to bloom. The black, purple, light cyan, and dark turquoise modules were negatively correlated with peduncle diameter, while the brown module was positively correlated with peduncle diameter.

#### Coexpression modules associated with specific genotypes at 0 DAI

Genotype and time to bloom were strongly correlated with several expression modules identified in tissues sampled at 0 DAI. These relationships may be representative of the initial transcript pool determining the cellular environment encountered by the fungus upon inoculation and can shape early host-pathogen interactions (Additional file 3).

##### Modules enriched for primary metabolism, ribosomal proteins, and phytohormone signal transduction were associated with bmr12 at 0 DAI

The magenta module was enriched for ribosomal proteins, amino acid biosynthesis, RNA transport, oxidative phosphorylation, and aminoacyl-RNA biosynthesis. This module includes large and small ribosomal proteins, eukaryotic initiation factors (eIF), F-type and V-type ATPase subunits, and NADPH dehydrogenase orthologs. Putative sulfoquinovosyltransferase Sobic.002G000600 was correlated with low average moisture and time to bloom.

The tan, black, and purple module eigengenes were negatively correlated with peduncle diameter. The black module contained several smaller lesion-associated genes, including a putative malate dehydrogenase (Sobic.001G073900). The black module, enriched for phytohormone signal transduction in JA, IAA and ABA pathways, was correlated with *bmr12* at 0 DAI. This corroborates the elevated levels of JA detected in *bmr12* plants at 3 DAI, though *bmr12* plants did not have elevated levels of IAA nor ABA. The purple module eigengene was additionally correlated with time to bloom.

##### Spliceosomal components were enriched in modules associated with the wild-type at 0 DAI

The yellow module was enriched for spliceosome (spliceosomal proteins and splicing factors) and RNA transport (nuclear pore complex proteins, eIF1, 4, 5). A probable protein-phosphatase 2C (PP2C)-like gene (Sobic.004G332900) is a potential susceptibility gene assigned to this module, which was positively correlated with a longer photoperiod at planting time and negatively correlated with time to bloom.

#### Meta-analysis: consensus associations with physiological traits

In order to assess traits across all three days, a meta-analysis was conducted in WGCNA. The dark turquoise module was constitutively correlated with the *bmr12* genotype (Fig. 7). It contained two AP2/ERF transcription factors, *SbEREB110* (Sobic.007G077300) and *SbEREB107* (Sobic.007G077001). Across all days, a flowering time (FT)-like ortholog (Sobic.010G164200) within the black module was strongly correlated with *bmr12*. Within the magenta module, putative sulfoquinovosyltransferase Sobic.002G00060 was correlated with *bmr12*. The pink, pale turquoise, brown, dark red, purple, black, and dark turquoise module eigengenes maintained a consensus association with plant physiological traits, including time to bloom and peduncle diameter (Additional file 3).

#### Cell wall and monolignol-related genes in response to abiotic and biotic stresses

The study of monolignol mutants requires attention to the impact of drought and pathogen infection on cell wall processes. The expression of many primary cell wall [38] and monolignol biosynthetic genes was correlated with fungal inoculations and/or water limitation (Additional file 3). Both water limitation treatment and *F. thapsinum* inoculation were strongly positively correlated with the expression of tricin synthase I (Sobic.007G218700, light green) and acetylserotonin O-methyltransferase I (Sobic.005G216100, red). Two yieldins (cell wall loosening proteins) (Sobic.002G055600, red, and Sobic.002G055700, red) were early genes (Table 2) correlated with *M. phaseolina* inoculation at 3 DAI. Phenolic biosynthetic genes associated with fungal infections included two eugenol O-methyltransferase orthologs (Sobic.007G058800, light green and Sobic.007G059100, red), and 4-coumarate-CoA ligase 1 (4CL1) (Sobic.007G089900, red). A glycogenin glucosyltransferase (Sobic.001G479800) and cellulose synthase (Sobic.001G021500) from the dark red module were constitutive lesion genes.

There were components of the cell wall that were also correlated with specific genotypes, highlighting the impact of monolignol biosynthetic mutations on diverse components of the cell wall (Additional file 3). An expansin (Sobic.003G112100, dark turquoise) was strongly positively correlated with the *bmr12* mutation and time to bloom, but negatively correlated with peduncle diameter. This expansin was also correlated with decreased levels of soluble syringic acid, syringaldehyde, and 4-coumaric acid, and elevated levels of soluble 4-hydroxybenzoic and sinapic acids. As previously observed, expression of the *Bmr12* and *Bmr6* genes (Sobic.007G047300, unassigned, and Sobic.004G071000, midnight blue, respectively) were reduced in lines with their respective mutant allele. However, expression levels of the *Bmr6* locus was positively correlated with the *bmr12* null allele. Notably, the expression of two CAD genes (Sobic.010G071800, cyan and Sobic.002G195400, red) were positively correlated with the *bmr6* mutation.

#### Transcription factors potentially involved in stress tolerance

A total of 583 transcription factors curated in the Grassius database were found to be expressed within the modules identified by WGCNA (Additional file 2). Selected transcription factors putatively involved in stress tolerance or cell wall are listed in Table 4. Nine transcription factors were assigned to modules in which their targets were predicted to be enriched (Additional file 2). Both heat shock transcription factors in the saddle brown module, *SbHSF4* (Sobic.001G243000) and *SbHSF11* (Sobic.002G271100), were positively correlated with the well-watered treatment. Their predicted targets were enriched for protein processing in the ER and for plant pathogen interactions. The predicted targets of *SbDOF23* (Sobic.006G267900, blue), a zinc finger transcription factor, were also in the blue module and were enriched for ubiquitin mediated proteolysis and with one carbon pool by folate. In the red module, *SbWRKY85* (Sobic.009G234100) is a transcription factor putatively involved in priming, and several genes from pathogenesis-related enriched categories were also early response or priming genes in this module (Additional file 4).

**Table 4:** Selected expressed transcription factors putatively involved in responses to pathogens and drought, and in priming

Grassius	Phytozome	Key trait(s)	Module Color	Defline
*SbWRKY23*	Sobic.003G000600	*M. phaseolina,* *F. thapsinum* inoculation; early response	Green	WRKY transcription factor 6
*SbEREB91*	Sobic.006G167800	*M. phaseolina,* *F. thapsinum* inoculation	Dark green	ethylene-responsive transcription factor 2
*SbWRKY75*	Sobic.008G060300	“	Green	probable WRKY transcription factor 70
*SbWRKY80*	Sobic.009G100500	“	Green	WRKY transcription factor 26
*SbEREB32*	Sobic.002G184400	*M. phaseolina* inoculation	Light yellow	ethylene-responsive transcription factor RAP2–4
*SbHB23*	Sobic.002G023900	*F. thapsinum* inoculation	Pink	homeobox protein rough sheath 1
*SbbZIP16*	Sobic.002G162800	“	Saddle brown	bZIP transcription factor 44
*SbbZIP17*	Sobic.002G225100	“	Turquoise	bZIP transcription factor TRAB1
*SbGLK40*	Sobic.010G224200	“	Green	probable transcription factor KAN4
*SbWHIRLY1*	Sobic.004G047500	*bmr6*	Grey60	single-stranded DNA-binding protein WHY2
*SbEREB107*	Sobic.007G077001	*bmr12*	Dark turquoise	NA (AP2-domain-containing)
*SbEREB110*	Sobic.007G077300	“	Dark turquoise	ethylene-responsive transcription factor 8
*SbbZIP38*	Sobic.003G363600	Constitutive lesion	Brown	transcription factor LG2
*SbGRAS25*	Sobic.003G377900	“	Pink	scarecrow-like protein 1
*SbZHD7*	Sobic.005G019800	“	Dark grey	NA
*SbGBP14*	Sobic.007G153700	“	Brown	probable transcription factor At4g00390
*SbOrphan149*	Sobic.009G126700	“	Orange	SNF2 domain-containing protein CLASSY 4
*SbMYB2*	Sobic.001G075300	Priming	Dark orange	myb-related protein Hv1
*SbWRKY85*	Sobic.009G234100	Priming	Red	probable WRKY transcription factor 34
*SbZIM2*	Sobic.001G100100	Smaller lesion	Turquoise	GATA transcription factor 19
*SbbHLH8*	Sobic.001G107400	“	Turquoise	transcription factor bHLH18-like
*N/A*	Sobic.001G435500	“	Turquoise	nuclear transcription factor Y subunit C-2-like
*SbbZIP16*	Sobic.002G162800	“	Saddle brown	bZIP transcription factor 44
*SbDOF19*	Sobic.004G266200	“	Midnight blue	dof zinc finger protein DOF3
*SbHB8*	Sobic.001G157400	Susceptibility	Yellow	NA
*SbARF26*	Sobic.008G169400	Susceptibility	Green-yellow	auxin response factor 25
*SbGLK7*	Sobic.002G016300	*bmr12*, drought treatment	Midnight blue	transcription factor HHO2

Transcription factors (TFs) potentially involved in coordinating defense to abiotic or biotic stresses include ethylene-responsive AP2/ERF, bZIP, WRKY, and bHLH TFs

#### Components of the mediator complex may be involved in the lesion response

The Mediator complex is the group of basal transcription factors that coordinate the interaction of RNA pol II with gene specific transcription factors. Originally discovered for its involvement in growth and development, the role of the Mediator complex in phenylpropanoid metabolism, immunity and abiotic stress tolerance has been of increasing interest [39–42]. In this study, several genes coding for Mediator subunits were associated with responses to *F. thapsinum* and *M. phaseolina* while others were correlated only with lesion length. *SbMed36a* (Sobic.004G349700, steel blue) was associated with *F. thapsinum* and, in tissues sampled at 3 DAI, with *M. phaseolina. SbMed14a* (Sobic.002G153700, pink) was positively correlated with *M. phaseolina* inoculation. Expression of *SbMed25a* (Sobic.002G164200, light green) was positively correlated with lesion length but not associated with fungal inoculation. *SbCyc1* (Sobic.002G256200, blue), the cyclin component of Mediator complex, was negatively correlated with lesion length in tissues sampled at 3 DAI and *SbMed30* (Sobic.006G051900, light green) was positively correlated with lesion length in tissues sampled at 3 DAI.

## Discussion

The objective of the current study was to examine the influence of cell wall modification on mechanisms of drought adaptation, and to identify potential mechanisms and pathways which promote or prevent lesion development during *M. phaseolina* or *F. thapsinum* infection*.*

The cell wall is a hub of stress surveillance and response, and the monolignol biosynthesis pathway is a component of a vastly interconnected metabolic grid whose manipulation can affect a broad array of other pathways [14]. The primary cell wall is principally made of polysaccharides (cellulose and hemicellulose), while lignin, a hydrophobic polymer composed of phenolic subunits, comprises the more rigid, structural secondary cell wall [18]. Complex layers of regulation coordinate lignin biosynthesis. It is irreversibly deposited as a function of developmental stage, response to abiotic stresses, interactions with microbes, and any synergy of these interactions. The role of cell wall integrity in immunity is far from straightforward, but cell wall alterations appear to allow the plant to renegotiate its position within the disease triangle (host susceptibility, pathogen host range, and environmental conditions) [17]. Mutants in cell wall biosynthesis across a number of systems display constitutively altered defense signaling, which has diverse effects on plant immunity that differ among pathosystems [43].

The reduction of lignin content is a key objective for cellulosic biofuel and forage production, necessitating an investigation into the robustness of reduced lignin lines to biotic and abiotic stresses. The *bmr* mutations in *CAD* and *COMT* genes, *bmr6* and *bmr12* (respectively), provide a valuable system to study the impact of lignin alteration on drought and disease response in sorghum [22, 23, 44]. Several studies have elucidated molecular mechanisms linked to drought tolerance in sorghum [45–49], making sorghum an opportune system to study mechanisms involved in drought response and their effects on disease response. Mounting evidence suggests that plants respond to simultaneous stresses in a manner that differs from simply the union or intersection of responses to stresses imposed individually [50–53].

Cell wall degradation is a component of many plant-fungal interactions, and cell wall integrity maintenance is crucial to plant homeostasis and adaptation. *M. phaseolina*, the necrotrophic causative agent of charcoal rot and one of the most destructive plant pathogens, encodes an extraordinarily high number of secreted cell wall-degrading enzymes that likely aid in host infection [54]. A number of *Fusarium* species have also been shown to encode cell wall degrading enzymes [55–57]. Although the genome sequence of *F. thapsinum* is not available, it is likely that *F. thapsinum* also encodes these enzymes based on its pathology and lifestyle, like other closely-related *Fusarium* species [58].

Disease control methods that can mitigate effects of environmental conditions, such as irrigation, may reduce stalk rot. However, sorghum is commonly grown on marginal lands under rain-fed conditions, in part due to its endogenous drought-tolerance. Resistance to stalk rot is a quantitative trait profoundly affected by environment [12, 59–61]. Specifically, QTLs associated with resistance to *F. thapsinum* and *M. phaseolina* explain a relatively small amount of disease resistance, ranging from 9 to 30%, and several loci are environment specific. The current findings support the consistent observations that despite decreased lignification, *bmr6* and *bmr12* plants are not more susceptible to the common stalk pathogens *F. thapsinum* and *M. phaseolina* under fungal pathogen infection, nor under combined pathogen and drought stresses. Unexpectedly, *bmr12* plants under water limitation had shorter lesions upon fungal inoculation than under well-watered conditions at 13 DAI (Fig. 3). This study confirms the validity of employing *bmr* mutants in breeding for stalk rot resistance and identifies other potential candidate pathways whose alteration could increase resistance.

**Fig. 8:**
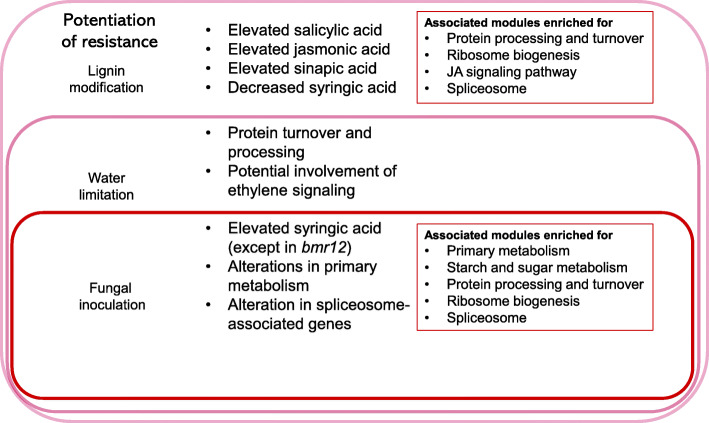
Proposed infection model: water limitation primes shorter lesion formation in *bmr12* plants. Lignin alteration in *bmr12* results in an altered hydroxycinnamic acid and soluble and cell wall bound phytohormone profile, which, combined with additional pathways associated with drought, may lead to increased disease resistance

The shorter lesion lengths observed in *bmr12* plants under water limitation suggests that water stress may prime a generalized defense response in this genotype [62]. Priming refers to the potentiation of defense without induction of a full defense response, leading a plant to be better prepared for a secondary stress [63]. This results in an earlier and stronger immune response upon pathogen challenge and can manifest in many combinations of mechanisms, including the production of bioactive metabolites, upregulation of defense genes, and callose deposition [63]. In the current study, *bmr12* plants were found to have elevated levels of SA and JA and an altered hydroxycinnamic acid profile compared to the wild-type. Thus, lignin modification may contribute to enhanced stress responses, conferring increased resistance. Water limitation may then result in altered flavonoids, ROS signaling, and ethylene signaling. These pathways may synergistically respond to disease (Fig. 8). Coexpression analysis was undertaken to further investigate these patterns.

Coexpression modules associated with fungal infection were enriched for pathways involved in primary metabolism, protein turnover, phenylpropanoid biosynthesis, and ETI components of plant-pathogen interaction. Genes involved in cell wall maintenance, including phenylpropanoid biosynthetic genes, laccases, and peroxidases, were involved in the early defense response. The combination of cell wall alteration and water-limitation may activate or overlap with downstream defense pathways that result in reduced lesion lengths. Several genes encoding drought response proteins, such as dirigent protein-like ortholog (Sobic.005G101600), were also correlated with water limitation. The expression of tricin synthase I (Sobic.007G218700) and acetylserotonin O-methyltransferase I (Sobic.005G216100) were correlated both with water limitation and with *F.thapsinum* inoculation. Flavonoids and phenylpropanoids are an important early component of diverse defense responses in plants [64–68]. In the current study, several priming genes encoding chalcone synthases were correlated with water-limitation, suggesting a potential role in drought-induced increased immunity. At 13 DAI, the majority of putative priming genes were associated with the water limitation treatment, as well as change in day length and time to bloom. Notably, these genes included Sobic.002G000600 (magenta), a sulfoquinovosyltransferase whose rice ortholog has flavonoid glycosylation activity [69, 70]. Overexpression of the rice sulfoquinovosyltransferase, *SQD2.1,* resulted in improved drought resistance. In the current study, putative sulfoquinovosyltransferase Sobic.002G000600 was correlated to *bmr12* and water limitation at 0 DAI. Several disease-responsive PR proteins and chitinases were coexpressed with these flavonoid biosynthetic enzymes, suggesting a coordinated response.

Other modules that were correlated with fungal infection at 3 DAI were enriched for ribosomal proteins, protein processing in the ER, ubiquitin-mediated proteolysis, and proteasome, highlighting the dramatic role of protein turnover and the increased synthesis of defensive enzymes and other defensive proteins in the pathogen response. Modules positively correlated to *bmr12* at 0 DAI were also positively correlated with *F. thapsinum* at 3 DAI, suggesting that these modules may have components that contribute to an earlier and more effective resistance response.

Plants with *bmr* mutations may be able to compensate for their lignin biosynthesis impairment with promiscuous orthologs. A Zrp4-like O-methyltransferase gene (Sobic.004G128400, red) was strongly correlated to *bmr12*, which might explain why sinapic acid is still produced in *bmr12* plants despite a hypothetical loss of ability to produce sinapoyl groups (Fig. 1). Likewise, two putative CAD genes (Sobic.010G071800, cyan and Sobic.002G195400, red) were associated with *bmr6*, which indicates a compensatory mechanism as was described in previous studies [23, 36].

*Fusarium thapsinum* infection resulted in elevated syringic acid levels in wild-type plants, but not in *bmr12* (as expected, as it is deficient in S-lignin biosynthesis; Fig. 1). This result suggests that syringic acid may be produced during response to *F. thapsinum* infection. Syringic acid has been identified as a potential virulence factor in *A. tumefaciens* C58C [71] and *Fusarium oxysporum* f. sp. *niveum* [72]. *Fusarium thapsinum* may commandeer syringic acid in wild-type plants, which is less abundant in *bmr12*, potentially affecting its ability to produce larger lesions in *bmr12* plants. Phenolic compounds can act as signaling molecules between plants and certain microorganisms [73], raising the possibility that disease resistance may be the result of signaling events surrounding phenylpropanoid metabolism in addition to the direct negative effect of these metabolites on fungal growth [35, 74, 75]. Modules enriched for stress responses including jasmonic acid signaling components, the spliceosome, and the peroxisome are positively correlated with *bmr12* (at 0 DAI). This corroborates the elevated levels of JA observed in *bmr12* plants at 3 DAI.

The *bmr12* tissues sampled at 3 DAI had elevated levels of JA and SA. JA and SA are signaling molecules with roles in defense whose pathways have been primarily understood to act antagonistically [76]. However, there have been cases of synergistic interactions in both monocots and dicots [77, 78]. For example, synergy between SA and JA has been previously described in barley, which resulted in the generation of reactive oxygen species (ROS) [79, 80]. In the current study, the expression of genes positively correlated with SA levels was generally not significantly correlated with JA levels, and vice versa. However, when genes were significantly correlated with both hormones, the correlations had the same sign (Additional file 2).

Pathogens are adept at manipulating plant hormone signals [81]. It has previously been reported that the host-derived IAA conjugate IAA-aspartate (IAA-Asp) aids in disease progression of both the bacterial pathogen *Pseudomonas syringae* and the fungal pathogen *Botrytis cinerea* in Arabidopsis but does not currently have a reported host function [82]. In the present study, there was a trend for higher IAA-Asp in plants inoculated with *F. thapsinum* than in plants inoculated with PDB, but it was not below the significance threshold of α = 0.1. Nonetheless, this trend suggests that IAA-Asp may play a role in *Fusarium* spp. pathogenesis on sorghum. In tissues sampled at 3 DAI, the modules positively correlated with IAA-Asp were enriched for pathways associated with protein turnover, including heat shock proteins and ubiquitin-mediated proteolysis. This result suggests that IAA-Asp may be involved in stress response by inducing heat shock proteins for protein stability. Heat shock transcription factors *SbHSF4* and *SbHSF11* were coexpressed with their predicted targets, which were enriched for heat shock proteins.

In addition to these defense and infection-related hormones, soluble phenolics have also been implicated as part of the priming response. Treatment of *Arabidopsis* with beta-aminobutyric acid (BABA), a known priming agent for disease resistance, was shown to result in altered levels of phenolic compounds including sinapic acid, primary metabolites, and the oxylipin-derived phytohormones OPDA and JA [83]. In the current study, 19 of the 34 putative priming genes were associated with soluble sinapic acid levels in tissues sampled at 3 DAI. Putative priming genes clustered with sinapic acid and *F. thapsinum* inoculation, and sinapic acid levels were elevated in *bmr12* compared to wild-type tissues. However, soluble sinapic acid levels were not elevated in *F. thapsinum*-infected tissues.

Drought conditions may prime defense pathways in *bmr12* plants. The diverse number of pathways that have been linked to defense priming in this study and in previous studies suggests that there may be numerous and interacting ways to activate and tune these pathways, and that drought may be an environmental trigger of priming in *bmr12* plants.

## Conclusion

Altering cell wall structure enables the study of the resultant metabolic and transcriptomic alterations on drought and disease response. Monolignol biosynthesis enzymes have been shown to interact with components of the immune system [84, 85]. In the current study, modifications in the monolignol biosynthesis pathway in *bmr12* plants impacted coexpression of genes involved in multiple pathways, including plant hormone signal transduction, RNA and protein processing and turnover, and transcription and translation. In particular, the coexpression patterns of primary and secondary cell wall biosynthetic genes and putative regulatory pathways that respond to both drought and disease point to the cell wall as the site of intricate connectivity between defense, growth, and development. Thus, drought conditions may have further primed *bmr12* plants for disease resistance through the activation of defense pathways. Monolignol mutant lines are not more susceptible and may even be hardier to some diseases, depending on environmental conditions. This method of activating pathways associated with priming may also be a way to increase disease resistance in crops while providing reduced lignin lines for bioenergy or forage production.

## Methods

### Seed availability

Seed of these genetic stocks are maintained and distributed by the USDA-ARS, Wheat, Sorghum, and Forage Research Unit, University of Nebraska, Lincoln, NE 68583–0937, and will be provided without cost to each applicant on written request. Genetic material of this release is deposited in the U.S. National Plant Germplasm System where it is available for research purposes, including development and commercialization of new varieties or cultivars. Released seed stocks are available upon request or through GRIN-Global.

### Growth conditions for well-watered and water-limited plants

Sorghum mutants *bmr6* and *bmr12*, near-isogenic to the wild-type, in the genetic background RTx430 were previously developed and are maintained by USDA-ARS, Lincoln, NE [86]. Greenhouse-grown seeds were planted at the University of Nebraska (UNL) Plant Growth facilities. Plants were grown year-round with supplemental high-pressure sodium lights in 25.4-cm-diameter pots containing a soil mixture with a 1:2:1:1 ratio of soil: peat moss: vermiculite: sand. Plants (one per pot) were arranged in a randomized split block design by watering conditions with eight replicates over time and watered with a fertilizer-water mixture according to experimental design. Water limitation was initiated when plants were in the boot stage (Fig. 2). Well-watered plants were watered daily while water-limited plants were watered only when soil moisture fell below 25% field capacity as measured with a 10HS Moisture Sensor (Decagon Devices) probe with a U30 Shuttle (Hobo). Water limitation continued from boot stage until tissue harvest. Each replicate consisted of 48 physiologically mature sorghum plants representing three genotypes (wild-type, *bmr6,* and *bmr12*), two watering conditions (well-watered and water-limited), three inocula (broth, *M. phaseolina*, or *F. thapsinum*), and three timepoints (0, 3, and 13 DAI). Eight such replicates were collected. The *bmr12* plants exhibited delayed bloom [87], resulting in some plants necessarily being culled from the experiment if they had not bloomed by 140 days after planting, because plant inoculations occurred at anthesis. Twenty-two *bmr12* plants, five *bmr6* plants, and no wild-type plants were culled from the experiment (Additional file 6).

### Inoculation, disease assessments and plant trait measurements

Toothpicks were incubated in batch culture at room temperature (22–23 °C) in potato dextrose broth (PDB) alone (the mock inoculation) or inoculated with agar discs (5 mm in diameter, one disc per 5 mL of PDB) from a fungal culture of *M. phaseolina* or *F. thapsinum* grown on one-half strength potato dextrose agar (PDA) medium for 4 days. The *F. thapsinum* isolate (H03-11S-9) was originally from a field in Lincoln, NE, and the *M. phaseolina* isolate (MP01–001) was a kind gift from G. Odvody (Texas A & M AgriLife Research and Extension Center, Corpus Christi). Plants were inoculated by producing a small wound on the peduncle, 5 cm below the base of the head, and inserting a fungus- or PDB-incubated toothpick into the wound [88]. Samples were collected at 0, 3, and 13 DAI with the following destructive assay: head lengths were measured, and heads were removed, peduncles were split down the middle longitudinally, then peduncle diameter and lesion length were measured.

#### Statistical testing for greenhouse data

Statistical testing for the greenhouse pathology data was conducted in the SAS programming environment using the PROC MIXED procedure for linear mixed models [89]. The model assessed the interaction of watering condition × inoculum × timepoint × genotype with replicate and replicate × water as random variables. The data were analyzed for heterogeneous variances using Levene’s test and adjusted appropriately using the REPEATED/GROUP = option. The script is available in Additional File 7.

### Sample collection for RNA-Seq and for metabolite analysis

From one-half of the split peduncle, 2-cm sections were harvested either surrounding the wound (if lesion was less than 20 mm) or from the base of the lesion (if lesion length equaled or exceeded 20 mm). Phenolics and phytohormones were assessed from a 2 cm peduncle section distal to the lesion (Fig. 2B and C).

This study was sequenced in two batches. In the first batch, the transcriptomes of wild-type, *bmr6*, and *bmr12* plants inoculated with *F. thapsinum* and *M. phaseolina*, and the PDB mock inoculation, were sequenced at 3 DAI. In the second batch, the study was expanded to include 0 and 13 DAI samples for wildtype, *bmr12*, *F. thapsinum*, and mock-inoculated plants. Because *bmr12* plants yielded unexpected results and as *M. phaseolina* is less commonly found on sorghum in Nebraska, *bmr6* and *M. phaseolina*-infected samples were not sequenced at 0 and 13 DAI. At least three biological replicates were sampled at 3 and 13 DAI in each unique condition (Fig. 2C, Additional File 1). Only two biological replicates per genotype × inoculation condition were sampled at 0 DAI, on the assumption that noise from sample harvesting would disguise signal from approximately 30 min of exposure to fungus, the time from inoculation to harvest. Figure 2 details the design of the greenhouse study, additionally clarifying the sampling procedure for subsequent analyses.

At 3 DAI, samples for assessment of phenolics and phytohormones were collected from a subset of *bmr12* and wild-type samples inoculated with *F. thapsinum* and PDB under both watering conditions (Fig. 2). Not all phytohormones were detected in all samples. Where phytohormones were not detected, the value of the limit of detection (LOD)/√2 was substituted where indicated (Additional file 1) after which the Wilcoxon rank-sum test was used to compare group means in the R programming environment. Substitute values were not plotted.

### Sample preparation

Peduncle samples for both RNA-Seq and metabolite analyses were ground in liquid nitrogen using a SPEX SamplePrep Freezer Mill 6870 (Metuchen, NJ, USA). For RNA, 50–100 mg of each sample was extracted using TRIzol reagent (Invitrogen, Carlsbad, CA) and purified with an RNA Clean and Concentrator kit (Zymo Research, Irvine, CA). The purified RNA samples were quantified using 260/280 ratios and 1–10 ng were sent to the University of Nebraska Medical Center Genomics Core Facility (https://www.unmc.edu/vcr/cores/vcr-cores/genomics/index.html) for further processing. RNA integrity was assessed at UNMC using an Agilent 2100 BioAnalyzer (Agilent, Santa Clara, CA). Libraries were constructed using the QuantSeq REV 3′ 96 barcode kit (Lexogen, Vienna, Austria) and were assayed for quality on the BioAnalyzer prior to pooling and sequencing. Library fragments were also analyzed by BioAnalyzer; quality control data (RIN and fragment size) is presented in Additional file 1 as the WGCNA ‘Traits’ matrix. In each run, samples were multiplexed across 4 lanes of a 75-cycle Illumina NextSeq 500 flow cell. The first sequencing run included samples collected on 3 DAI, and the second sequencing run included additional PDB inoculated samples within 3 DAI. Wild-type and *bmr12*, well-watered and water limited, mock-inoculated and *F. thapsinum*-inoculated samples on both 0 and 13 DAI were sequenced in Run 3. Because the runs were sequenced in batches, and not every condition was replicated in every batch, the separation between the 3 DAI samples and the 0 and 13 DAI samples is confounded with the sequencing run. This is reflected in the clustering pattern of the samples on a PCA plot (Additional file 8). Separation is more clearly seen when the plots are presented by DAI. A consensus network was constructed from samples analyzed by timepoint in order to minimize these batch effects. Sequence data were submitted to SRA under BioProject PRJNA573931. Alignment statistics can be found in Additional File 14.

### RNA-Seq data cleaning and alignment

Barcodes were removed and the 132-bp adapters trimmed with a Lexogen-recommended script (Additional file 9). Reads were pseudoaligned to the *S. bicolor* genome (v3.1) [90] downloaded from Phytozome [91] using kallisto v45 (Additional file 10) [92]. Kallisto .hd5 files were read into the R programming environment with tximport (Additional file 10). The qPCR analysis indicated agreement with RNA-Seq findings (Additional files 15 and 16) [93].

Pairwise differential expression testing was performed in DESeq2 but resulted in < 20 DE genes under each condition tested. The code for performing these comparisons is included in Additional file 11.

### Network analysis

Reads were pre-filtered to genes containing cpm > 10 and transformed with the native variance stabilizing transformation in DESeq2, as recommended by the authors of WGCNA [94, 95]. A consensus network was constructed for gene expression across the three timepoints (Additional file 11). Signed networks were constructed by DAI using Pearson correlation in WGCNA in a modification of the second procedure described in WGCNA tutorials (https://horvath.genetics.ucla.edu/html/CoexpressionNetwork/Rpackages/WGCNA/Tutorials/), modified for three groups [94, 95]. Module-trait Pearson correlation was calculated and adjusted for false discovery rates (FDR) using the Benjamini-Hochberg (BH) method [96–98].

### Gene set analysis

KEGG enrichment in modules identified through WGCNA was calculated using Fisher’s exact test in KOBAS (http://kobas.cbi.pku.edu.cn/) adjusted for FDR with BH [99, 100]. TF enrichment was calculated using PlantTFDB (http://planttfdb.cbi.pku.edu.cn/) [101].

### Analysis of secondary metabolites

Phytohormone analysis was conducted at the UNL Proteomics and Metabolomics Facility following procedures described previously [102–104].

Phenolic analysis was conducted as described previously [28] with modifications for detection with an Agilent 7890B gas chromatograph with 5977A mass spectrometer integrated system as described in Additional file 12.

Metabolite analysis was performed in the R programming environment (3.6.1) (Additional file 13).

## Supplementary Information

**Additional file 1.** Data table containing all plants sampled for this study, including metabolite samples, where applicable.

**Additional file 2.** Filterable table of module-trait correlations for lesion-related genes, as defined in the text.

**Additional file 3.** Module-trait correlation for all modules calculated by WGCNA.

**Additional file 4.** KEGG enrichment results for all modules, calculated by the KOBAS tool.

**Additional file 5.** Relative expression of module eigengenes, calculated by WGCNA.

**Additional file 6 **Time to bloom per genotype. The *bmr12* mutant plants exhibit delayed bloom, resulting in their disproportionate culling from the experiment, as plants were inoculated after bloom, resulting in disproportionate missing data from *bmr12* plants.

**Additional file 7.** SAS script to calculate a linear model to compare lesion lengths.

**Additional file 8.** Principle component analysis (PCA) of samples indicating significant separation by day of sampling (A), which is confounded with RNA sequencing run, and little separation based on water treatment (B). Separated by day of run (C), the separation between treatment conditions is clarified.

**Additional file 9.** Lexogen-recommended script for trimming raw RNA-Seq reads.

**Additional file 10 **Shell script for using Kallisto to pseudoalign reads to the *S. bicolor* genome.

**Additional file 11.** R markdown notebook containing WGCNA and downstream analysis.

**Additional file 12.** GC/MS protocol, adapted from Palmer et al. 2008.

**Additional file 13.** R markdown notebook containing time to bloom and metabolite analysis.

**Additional file 14.** Pseudoalignment statistics from Kallisto v0.44.

**Additional file 15.** Excel workbook of qPCR correlation analysis.

**Additional file 16.** Complete Cq values for qPCR validation.

## Data Availability

Raw data from greenhouse work is available in the additional files of this manuscript. Sequence data has been submitted to SRA under BioProject PRJNA573931. All scripts and R notebooks used for data analysis are also available in the additional files of this manuscript, and at https://github.com/khasinwsfru/bmr-drought.

## Abbreviations

ABA: Abscisic acid
Bmr: Brown midrib
CAD: Cinnamyl alcohol dehydrogenase
CCoAOMT: Caffeoyl-CoA O-methyltransferase
CCR: Cinnamoyl-CoA reductase
COMT: Caffeic acid O-methyltransferase
GA19: Gibberellin A19
GA53: Gibberellin A53
DAI: Days after inoculation
IAA: Indoleacetic acid
IAA-Asp: Indoleacetic acid-aspartate
JA: Jasmonic acid
OPDA: 12-oxo-phytodienoic acid
PAL: Phenylalanine ammonia lyase
PDB: Potato Dextrose Broth
SA: Salicylic acid
WGCNA: Weighted gene correlation network analysis
