# Supplementary material for: Pathogen and drought stress affect cell wall and phytohormone signaling to shape host responses in a sorghum COMT bmr12 mutant
Source: BMC Plant Biol. 2021 Aug 21;21:391. doi: 10.1186/s12870-021-03149-5 (PMC8379876; doi:10.1186/s12870-021-03149-5)

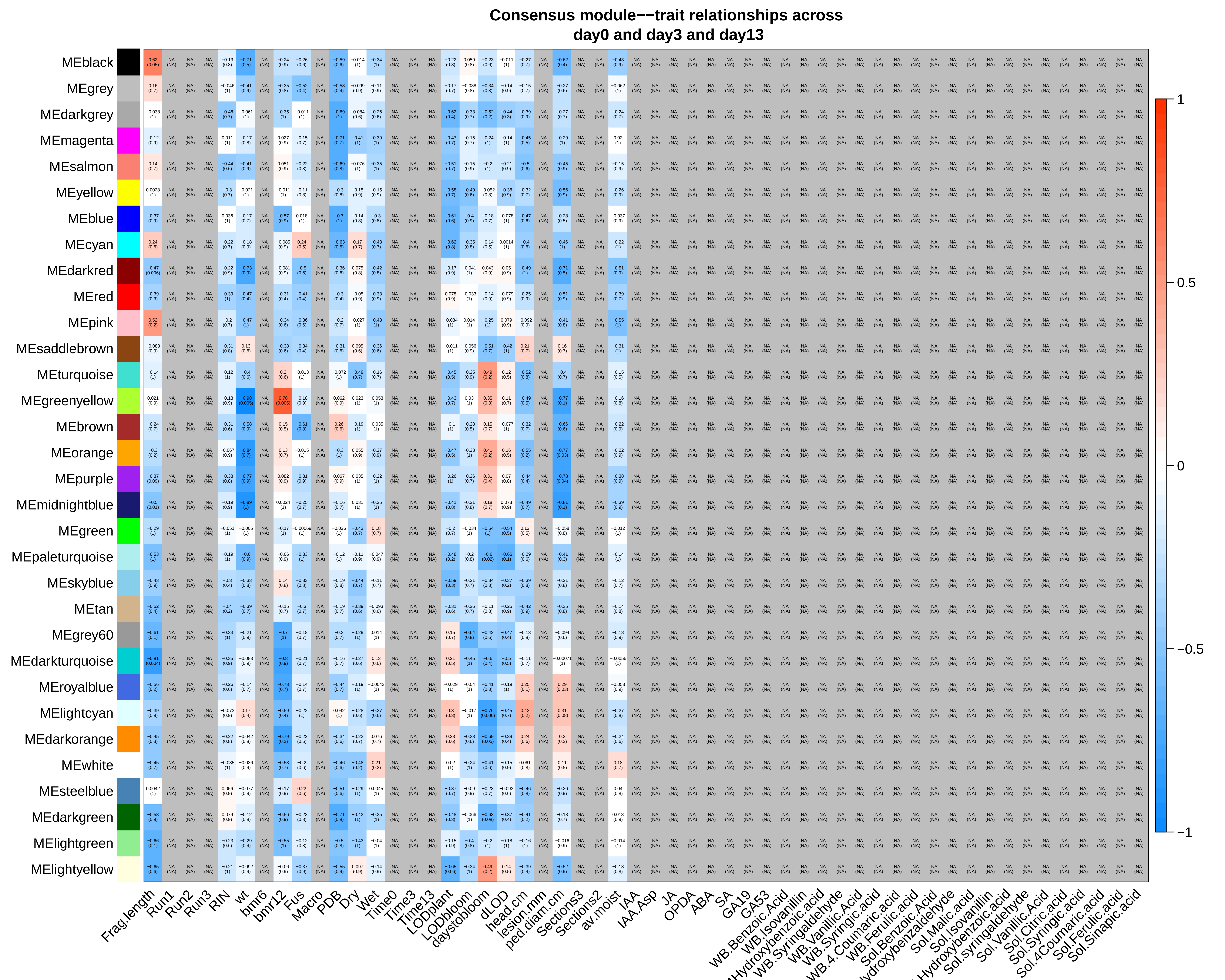
Consensus module−−trait relationships across
day0 and day3 and day13
MEblack
MEgrey
MEdarkgrey
MEmagenta
MEsalmon
MEyellow
MEblue
MEcyan
MEdarkred
MEred
MEpink
MEsaddlebrown
MEturquoise
MEgreenyellow
MEbrown
MEorange
MEpurple
MEmidnightblue
MEgreen
MEpaleturquoise
MEskyblue
MEtan
MEgrey60
MEdarkturquoise
MEroyalblue
MElightcyan
MEdarkorange
MEwhite
MEsteelblue
MEdarkgreen
MElightgreen
MElightyellow
Frag.length
Run1
Run2
Run3
RIN
wt
bmr6
bmr12
Fus
Macro
PDB
Dry
Wet
Time0
Time3
Time13
LODplant
LODbloom
daystobloom
dLOD
head.cm
lesion.mm
ped.diam.cm
Sections3
Sections2
av.moist
IAA
IAA.Asp
JA
OPDA
ABA
SA
GA19
GA53
WB.Benzoic.Acid
WB.Isovanillin
WB.Syringaldehyde
WB.Vanillic.Acid
WB.Syringic.acid
WB.4.Coumaric.acid
WB.Ferulic.acid
Sol.Benzoic.Acid
Sol.Malic.acid
Sol.Isovanillin
Sol.syringaldehyde
Sol.Vanillic.Acid
Sol.Citric.acid
Sol.Syringic.acid
Sol.4Coumaric.acid
Sol.Ferulic.acid
Sol.Sinapic.acid
1
0.5
0
−0.5
−1

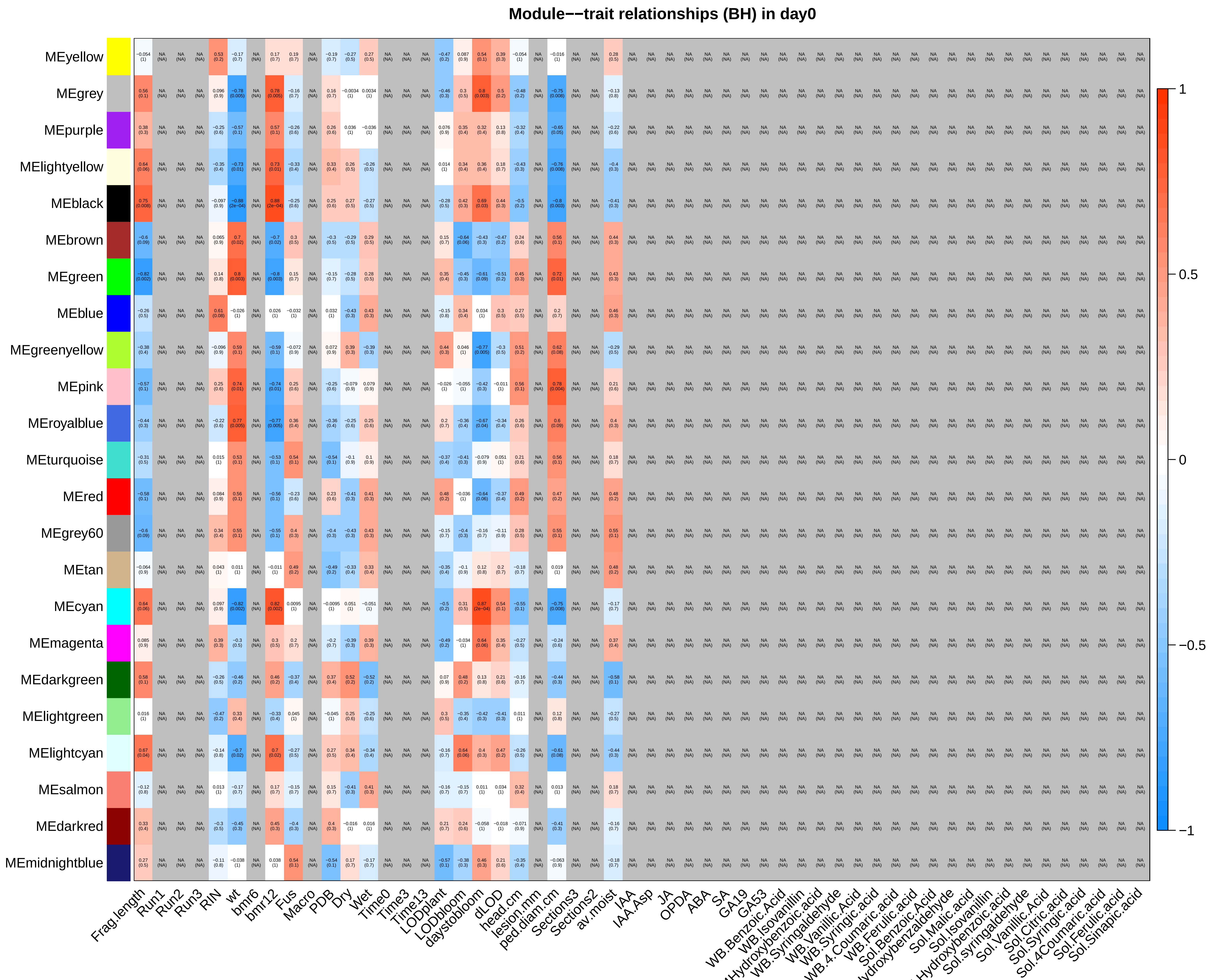
Module−−trait relationships (BH) in day0
MEyellow
MEgrey
MEpurple
MElightyellow
MEblack
MEbrown
MEgreen
MEblue
MEgreenyellow
MEpink
MEroyalblue
MEturquoise
MEred
MEgrey60
MEtan
MEcyan
MEmagenta
MEdarkgreen
MElightgreen
MElightcyan
MEsalmon
MEdarkred
MEmidnightblue
Frag.length
Run1
Run2
Run3
RIN
wt
bmr6
bmr12
Fus
Macro
PDB
Dry
Wet
Time0
Time3
Time13
LODplant
LODbloom
daystobloom
dLOD
head.cm
lesion.mm
ped.diam.cm
Sections3
Sections2
av.moist
IAA
IAA.Asp
JA
OPDA
ABA
SA
GA19
GA53
WB.Benzoic.Acid
WB.Isovanillin
WB.Syringaldehyde
WB.Vanillic.Acid
WB.Syringic.acid
WB.4.Coumaric.acid
WB.Ferulic.acid
Sol.Benzoic.Acid
Sol.Malic.acid
Sol.Isovanillin
Sol.syringaldehyde
Sol.Vanillic.Acid
Sol.Citric.acid
Sol.Syringic.acid
Sol.4Coumaric.acid
Sol.Ferulic.acid
Sol.Sinapic.acid
1
0.5
0
−0.5
−1

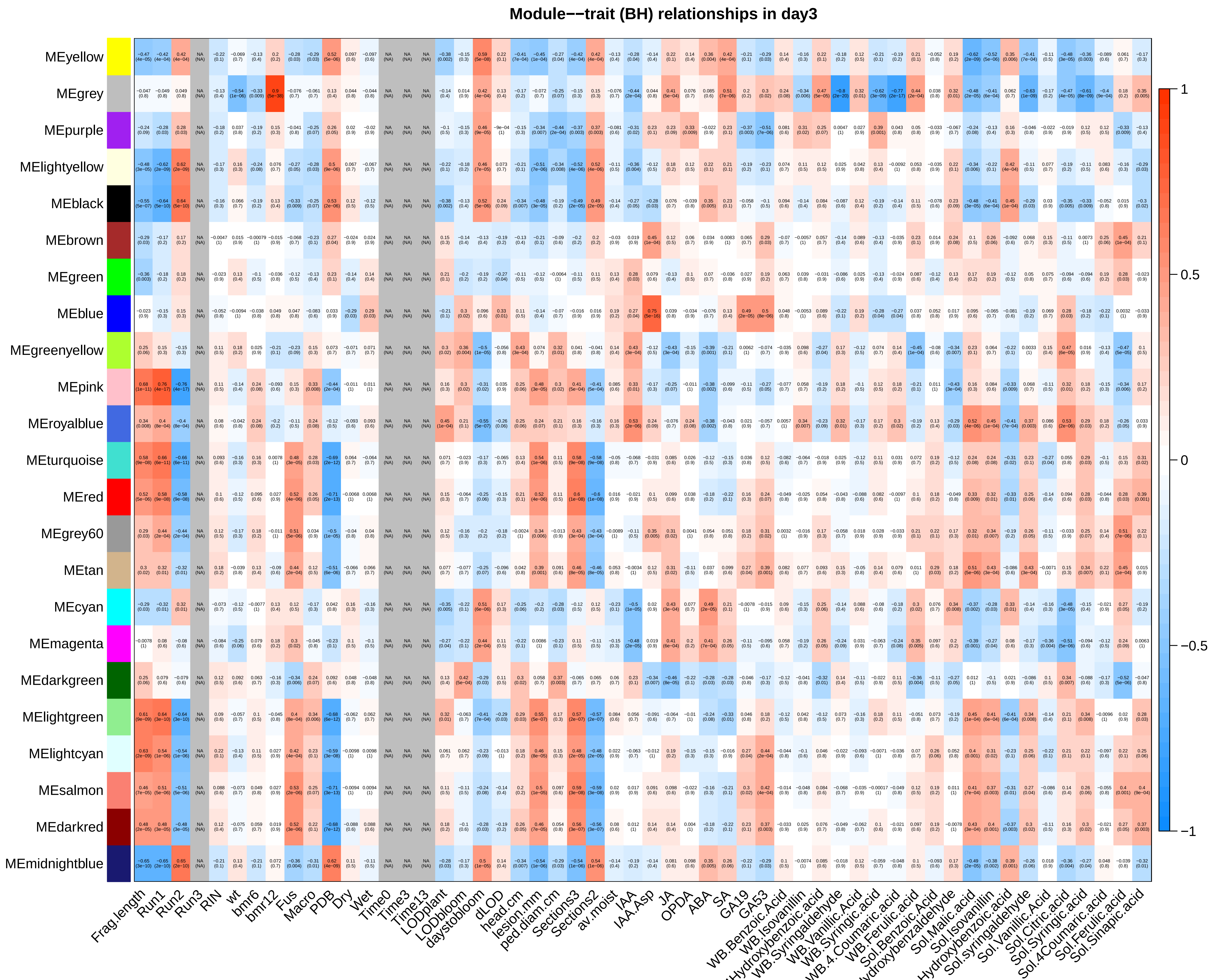
Module−−trait (BH) relationships in day3
MEyellow
MEgrey
MEpurple
MElightyellow
MEblack
MEbrown
MEgreen
MEblue
MEgreenyellow
MEpink
MEroyalblue
MEturquoise
MEred
MEgrey60
MEtan
MEcyan
MEmagenta
MEdarkgreen
MElightgreen
MElightcyan
MEsalmon
MEdarkred
MEmidnightblue
Frag.length
Run1
Run2
Run3
RIN
wt
bmr6
bmr12
Fus
Macro
PDB
Dry
Wet
Time0
Time3
Time13
LODplant
LODbloom
daystobloom
dLOD
head.cm
lesion.mm
ped.diam.cm
Sections3
Sections2
av.moist
IAA
IAA.Asp
JA
OPDA
ABA
SA
GA19
GA53
WB.Benzoic.Acid
WB.Isovanillin
WB.Syringaldehyde
WB.Vanillic.Acid
WB.Syringic.acid
WB.4.Coumaric.acid
WB.Ferulic.acid
Sol.Benzoic.Acid
Sol.Malic.acid
Sol.Isovanillin
Sol.syringaldehyde
Sol.Vanillic.Acid
Sol.Citric.acid
Sol.Syringic.acid
Sol.4Coumaric.acid
Sol.Ferulic.acid
Sol.Sinapic.acid
1
0.5
0
−0.5
−1

**Module−−trait relationships (BH) in day13**

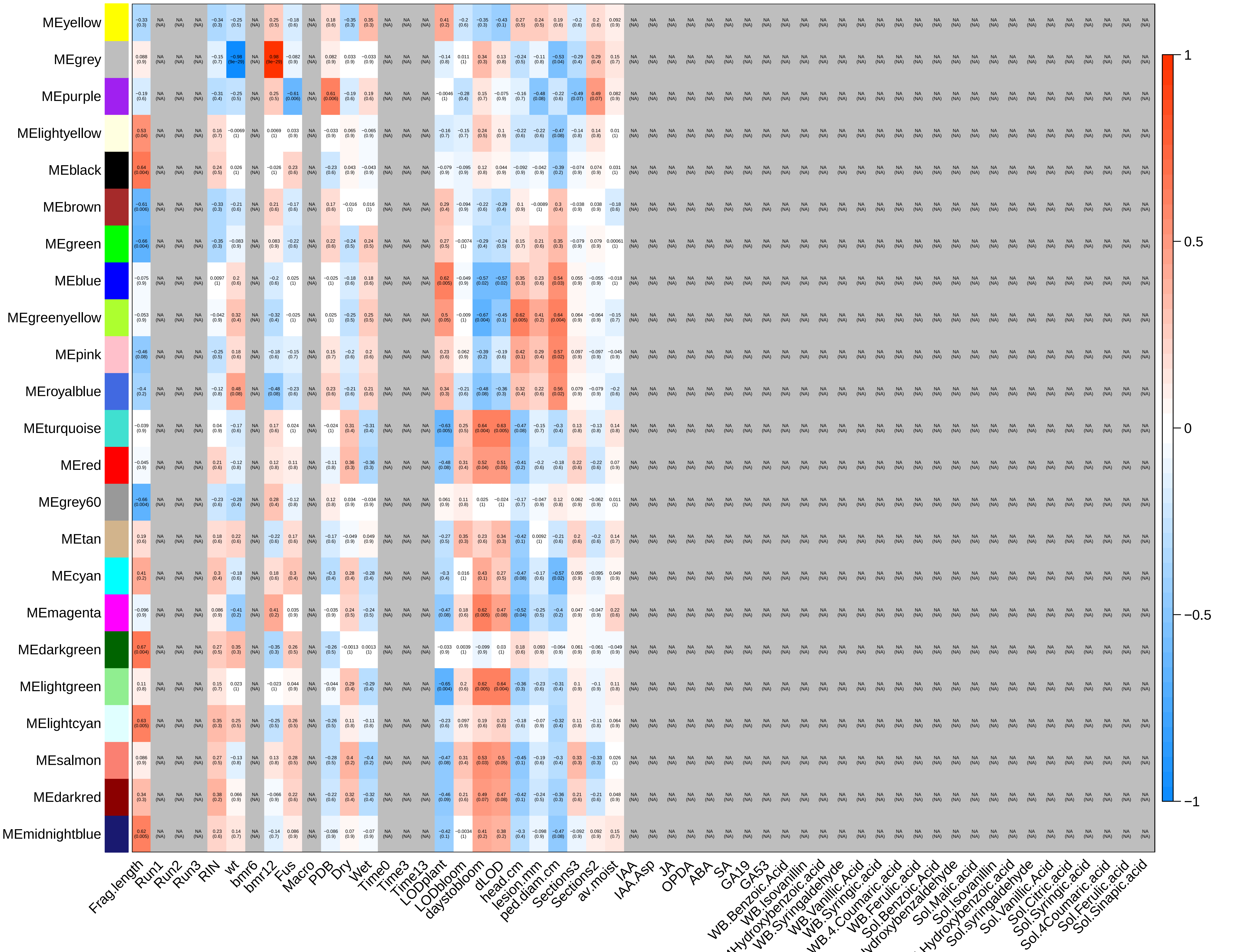

Supplement: Supplementary file 3 — Additional file 3. Module-trait correlation for all modules calculated by WGCNA. [file 12870_2021_3149_MOESM3_ESM.pdf]
