# Supplementary figures and images for: Pathogen and drought stress affect cell wall and phytohormone signaling to shape host responses in a sorghum COMT bmr12 mutant
Source: BMC Plant Biol. 2021 Aug 21;21:391. doi: 10.1186/s12870-021-03149-5 (PMC8379876; doi:10.1186/s12870-021-03149-5)

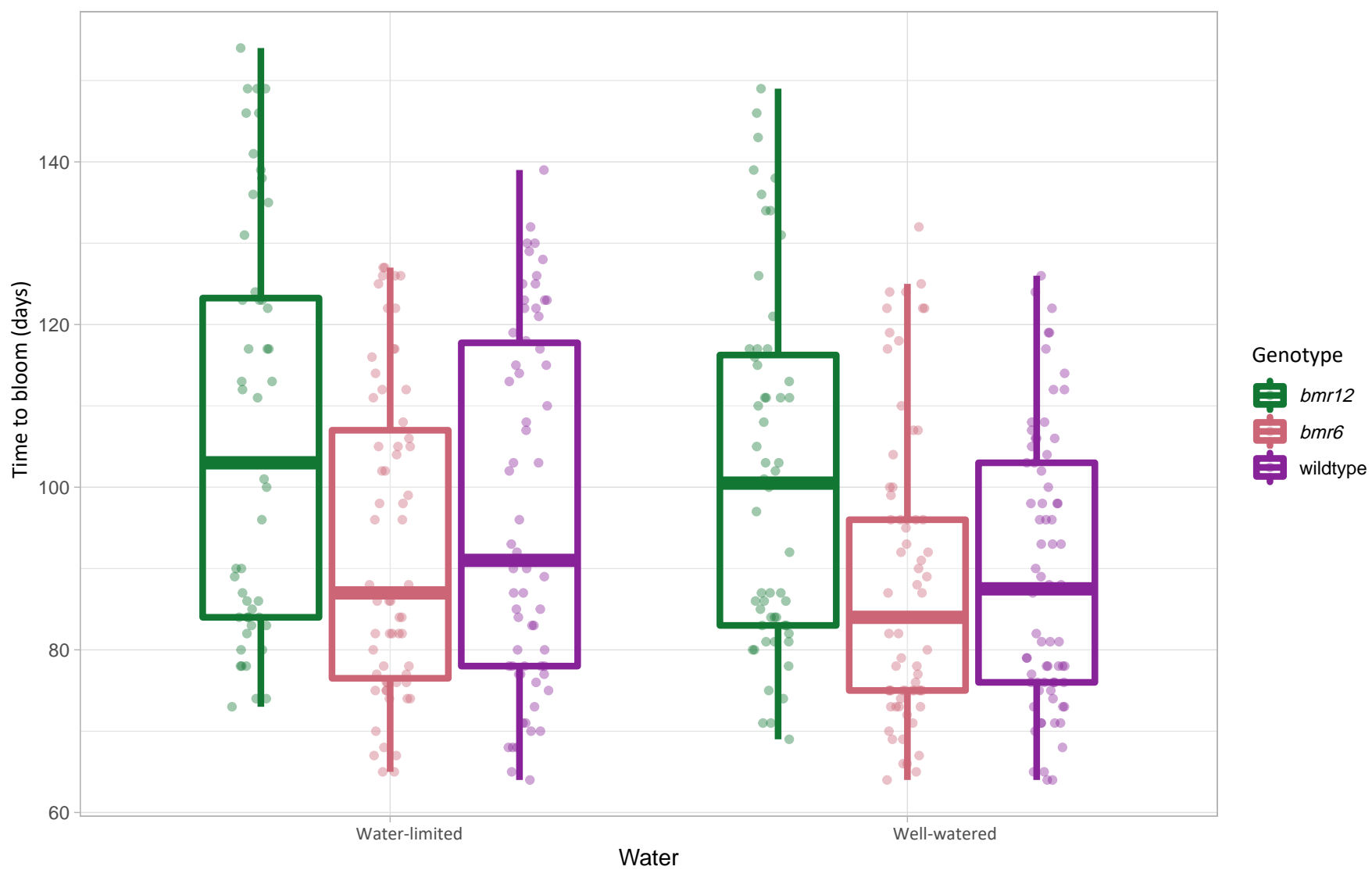

Supplement: Supplementary file 6 — Additional file 6 Time to bloom per genotype. The bmr12 mutant plants exhibit delayed bloom, resulting in their disproportionate culling from the experiment, as plants were inoculated after bloom, resulting in disproportionate missing data from bmr12 plants. [file 12870_2021_3149_MOESM6_ESM.pdf]

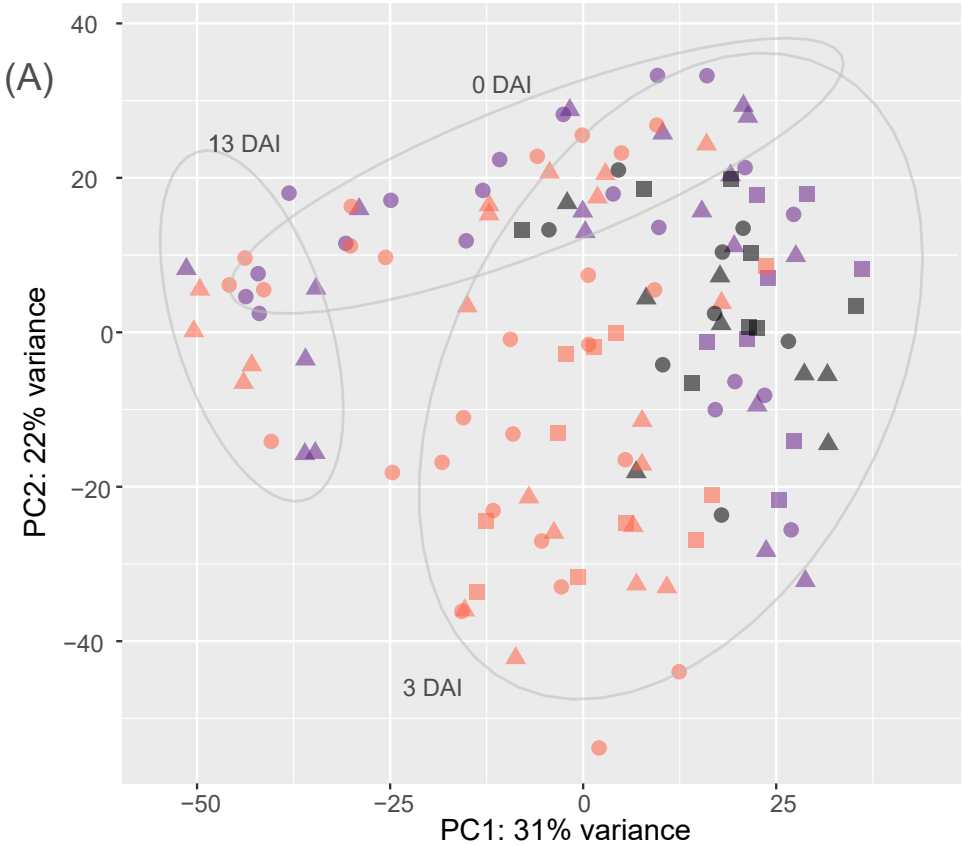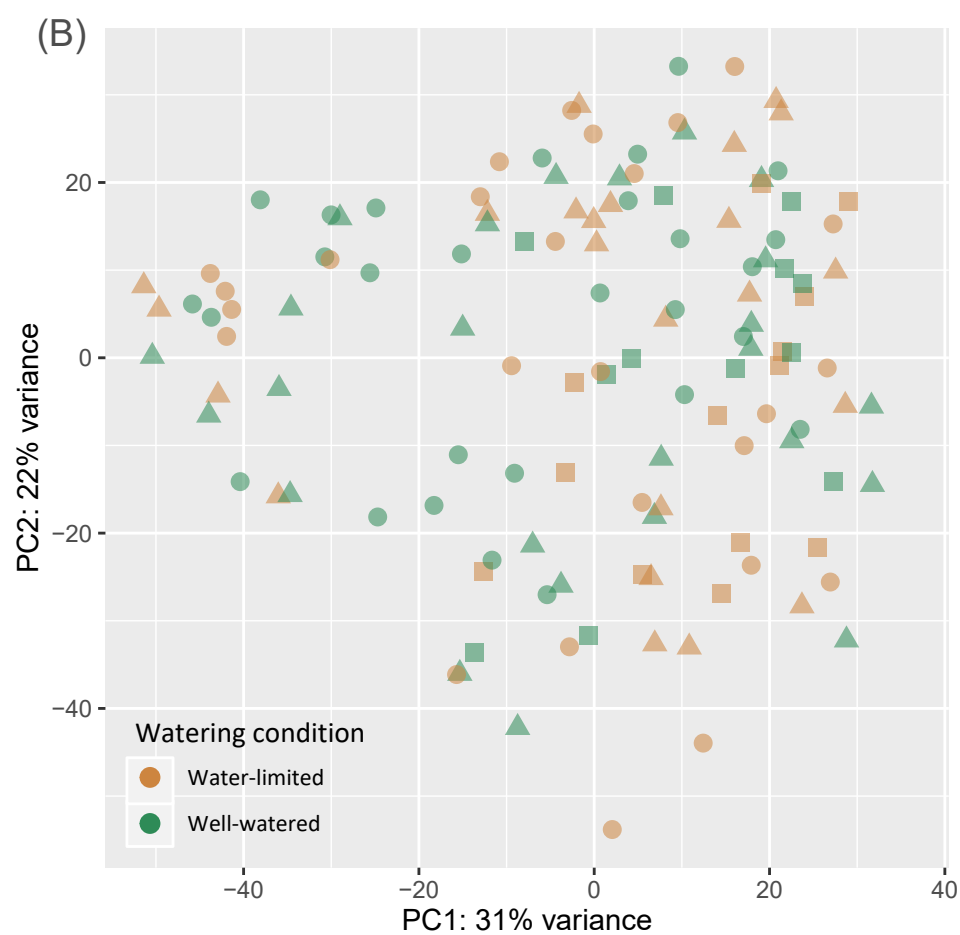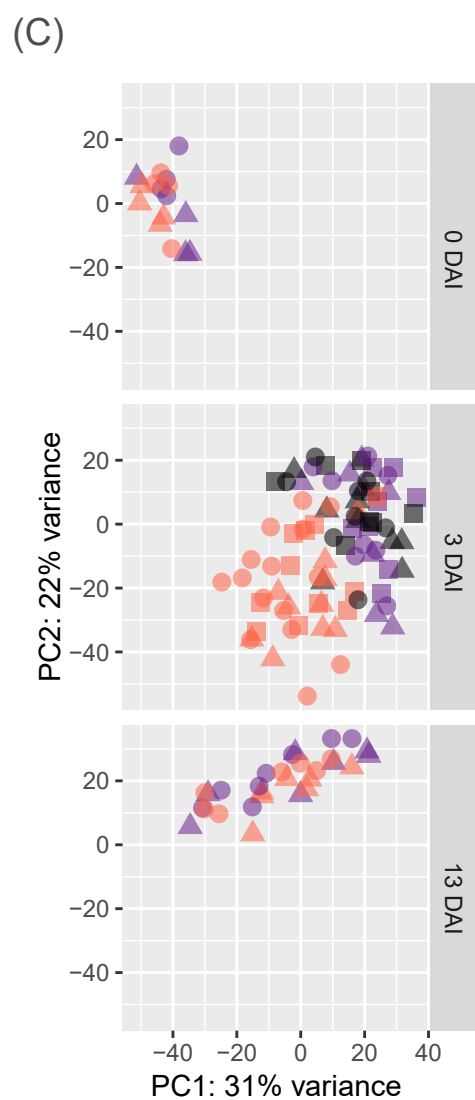

Supplement: Supplementary file 8 — Additional file 8. Principle component analysis (PCA) of samples indicating significant separation by day of sampling (A), which is confounded with RNA sequencing run, and little separation based on water treatment (B). Separated by day of run (C), the separation between treatment conditions is clarified. [file 12870_2021_3149_MOESM8_ESM.pdf]
