# Supplementary material for: Pathogen and drought stress affect cell wall and phytohormone signaling to shape host responses in a sorghum COMT bmr12 mutant
Source: BMC Plant Biol. 2021 Aug 21;21:391. doi: 10.1186/s12870-021-03149-5 (PMC8379876; doi:10.1186/s12870-021-03149-5)
Supplement: Supplementary file 12 — Additional file 12. GC/MS protocol, adapted from Palmer et al. 2008. [file 12870_2021_3149_MOESM12_ESM.docx]

USDA-ARS, Biomass Research Laboratory

Lincoln, NE

Soluble/Wallbound Prep with Base Hydrolysis: Fresh Tissue

NP, Version 1.0 6/24/2005 Updated by EDS, Version 2.0 12/9/2015

Updated by LFB, Version 3.0 2-5-19

Preparation of samples for analysis by GC/MS.

Personal Protective Equipment (PPE) and Safety and Health Procedures: **Read MSDS sheets located in 365 Plant Science for all chemicals and solutions used.** PPE includes lab coat, safety glasses, gloves, fume hood.

Equipment

Vortex

Centrivap

Tabletop centrifuge

Chemicals

4.0 N NaOH

6.0 M HCl

Ethyl Acetate

1.5% Acetic Acid in 50% MeOH

Acetone

4-methyl cinnamic acid

Ferulic acid

α-methyl cinnamic acid

Toluic acid

**Bold** 🡪 Safety and Health Procedure

*Italics* 🡪 Quality Control Procedure

Sample Preparation and Extraction

1. Weigh out 50 mg of ground, fresh frozen tissue into a 2 ml microfuge tube.
2. Extract with 1.3 ml of 1.5% acetic acid in 50% methanol containing 0.0078 mg/mL 4-methyl cinnamic acid* by vortexing briefly and then inverting continuously for 1 hour.
3. Centrifuge at 14,000 RPM for 5 minutes. Pipette the supernatant into a new 2 ml tube, save the pellet.
4. Add 0.5 ml of 1.5% acetic acid in 50% methanol with NO internal standard to the pellet, vortex to mix and repeat step 3.
5. Wash the pellet with 0.5 ml of acetone, centrifuge at 14,000 RPM for 5 minutes, remove excess acetone and spin to dryness in the centrivap in the hood. Alternatively, samples can be dried overnight in the hood. Dried pellets can be stored at -20°C until ready for use.
6. Centrifuge the supernatant again at 14,000 RPM for 5 minutes to spin down particulates. Transfer the supernatant to a fresh microfuge tube and spin to dryness in the centrivap.

*subject to change depending on amount dried for derivatization.

Base Hydrolysis: Soluble

1. Add 400 µL of 4M NaOH to the dried supernatant from step 5. For every batch (soluble and wall-bound), add 20-25 mg of ferulic acid to a pre-weighed tube, add 400 µL of 4.0 M NaOH and treat as a normal sample. Vortex until mixed thoroughly. Incubate at 90 °C for 2 hours. Gently invert tubes ~ every 30 minutes.
2. Acidify by adding 350 µL of 6.0 M HCl, vortex thoroughly, and verify acidity with Litmus paper. The acidity needs to be pH of 2.0 or less (deep red).

Base Hydrolysis: Wall-Bound

1. Make a solution of 5 mg/mL α-methyl cinnamic acid in 100% MeOH. Using this stock solution, make a base hydrolysis solution (4M NaOH with 0.5 mg/mL α-methyl cinnamic acid*).
2. Add 400 µL of the base hydrolysis solution and 200 µL milliQ water to the dried pellet from step 4 of the extraction. Briefly centrifuge to spin down all debris into solution.
3. For every batch (soluble and wall-bound), add 25 mg of ferulic acid to a pre-weighed tube, add 400 µL of 4.0 M NaOH and treat as a normal sample. Vortex briefly and incubate at 90 °C for 2 hours. Gently invert tubes ~ every 30 minutes.
4. Acidify by adding 350 µL of 6.0 M HCl, vortex thoroughly, and verify acidity with Litmus paper. The acidity needs to be pH of 2.0 or less (deep red).

*subject to change depending on amount dried for derivatization.

Ethyl Acetate Back Extraction

1. Add 500 µL of Ethyl acetate. Mix well by vortexing for 30 seconds.
2. Centrifuge at 14,000 RPM for 5 minutes.
3. Carefully remove ethyl acetate layer and add to a new 2 ml tube.
4. Repeat ethyl acetate extraction 2 more times, pooling all three ethyl acetate extractions.
5. Dry pooled ethyl acetate in the centrivap in the hood. Alternatively, samples can be dried in the hood overnight. If there is still residual ethyl acetate the following day, samples can be dried in the centrivap for 30 minutes.
6. Weigh the dried ferulic acid sample and compare to the initial weight. This verifies successful back extraction.

Derivitization:

1. Make a solution containing 0.25 mg/mL toluic acid in 100% methanol.
2. Resuspend wall-bound extracts in 1.0 mL methanol/toluic acid solution from step 1.
3. Make a solution containing 0.0039 mg/mL toluic acid in 100% methanol (or make a 1: dilution of solution from step 1).
4. Resuspend soluble extractions in 0.5 mL methanol/toluic acid from step 3.
5. For wall-bound extracts, add 20 µL of each sample to GC vial. **Dry it down completely in the hood.**
6. For soluble extracts, add 200 µL of each sample to a GC vial. **Dry it down completely in the hood.**
7. Add 50 µL pyridine to each GC vial.
8. Add 80 µL MSTFA derivatization reagent to each sample.
9. Mix and incubate in water bath at 60 °C for 90 minutes. Keep lids above water.
10. Remove GC vials from water bath and dry off the lids if necessary.
11. Transfer the derivatization reaction to a GC insert.
12. Run on GC (thioacidolysis protocol).
13. After resuspending in methanol and taking aliquot for derivitization, leave sample tubes opened in hood and allow residual methanol to evaporate. If samples need to be rederivitized in the future, bring up to volume in 1.0 mL methanol with no standards.

*Internal standards (4-methyl cinnamic acid for soluble and α-methyl cinnamic acid for wall-bound) should have a final mass of 4µg in the derivitization.*

Helpful tips:

It works best to break up the protocol across three days as follows:

Day 1: Sample Preparation and Extraction. Drying the 50:50 MeOH:H_2_O can take several hours in the centrivap and an overnight stint in the hood helps.

Day 2: Base Hydrolysis and Ethyl Acetate Back Extraction.

Day 3: Derivitization and Injection.

I recommend using a repeater pipette for adding solutions to each tube quickly and accurately. The GC can process 36 samples every 24 hours. Batch sizes should be adjusted accordingly.
