# Supplementary material for: Pathogen and drought stress affect cell wall and phytohormone signaling to shape host responses in a sorghum COMT bmr12 mutant
Source: BMC Plant Biol. 2021 Aug 21;21:391. doi: 10.1186/s12870-021-03149-5 (PMC8379876; doi:10.1186/s12870-021-03149-5)
Supplement: Supplementary file 15 — Additional file 15. Excel workbook of qPCR correlation analysis. [file 12870_2021_3149_MOESM15_ESM.docx]

qPCR validation of RNA-Seq data[1]

1. Experimental design

Plates run, per gene:

**(No-RT control)**

1. **Samples**
   Samples were ground and processed as described in Materials and Methods, as the qPCR was repeated on these samples.
2. **Reverse transcription**

Reverse transcription was conducted from 500 ng of RNA according to the following protocol from the Roche Transcriptor First Strand cDNA synthesis kit (04379012001):

Per reaction:

500 ng RNA + water to 10 uL

oligo-dT primer: 1 uL

random hexamer primers: 2 uL

This reaction was incubated for 10 min at 65 degrees C and then placed on ice.

After incubation on ice, the following was added to each reaction:

4 uL reverse transcriptase buffer

0.5 uL Protector RNase inhibitor

2 uL 10 mM dNTPs

0.5 uL reverse transcriptase

**Reverse transcription conditions:**

25 C x 10 min

55 C x 30 min

85 C x 5 min

Upon completion of reverse transcription, 80 uL of DEPC treated water was added to each reaction, to a total volume of 100 uL. Samples were stored at -20 C.

1. **qPCR target information**

Primers targeting the phenylpropanoid pathway have been described [2, 3]. This paper used the geometric mean of two other reference genes below, identified due to the stability in their expression levels under both biotic and abiotic stresses [4, 5].

| **PrimerID** | **Description** | **Accession** | **PrimerF** | **TM_F** | **PrimerR** | **TM_R** | **Product** |
| --- | --- | --- | --- | --- | --- | --- | --- |
| SAND | SAND family protein | XM_021457233 | TCCACTGGCTGATAAGACTCAAGC | 62.43 | GGTACTGCCTGTGTCGGTACAAGT | 64.38 | 204 |
| 18S | 18s rRNA | AH001770 | CGGGCGCGTTAGTGTCTGGT | 65.18 | CGATCCCTGGTCGGCATCGT | 64.47 | 227 |

1. **qPCR reaction conditions**

In triplicate, the following was added to each well:

10 uL Bio-Rad Luna 2x Universal qPCR Master Mix (M3003S)

1 uL 10 uM Primer F

1 uL 10 uM Primer R

1 uL cDNA (18S, SAND, 4CL) or 4 uL cDNA (CAD, PAL, C3H)

7 uL water (18S, SAND, 4CL) or 4 uL water (CAD, PAL, C3H)

Cycling conditions were as described by Tetrealt () and Scully () on a Bio-Rad CFX96 instrument. Cqs were detected using the BioRad CFX software.

1. **Validation**

Phenylpropanoid primers were validated as described previously. New reference primers were validated by melt curve analysis. (Primer dimer signatures come from no-template controls; one sample with an improper melt curve was removed from analysis.)


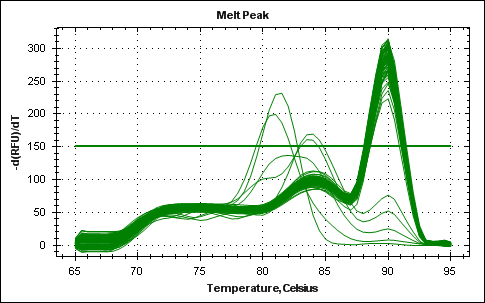

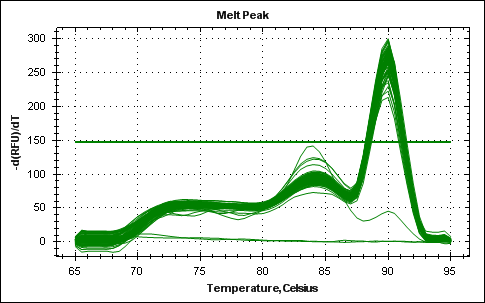


18S – well-watered 18S – dry


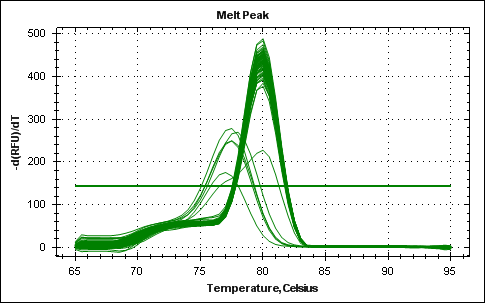

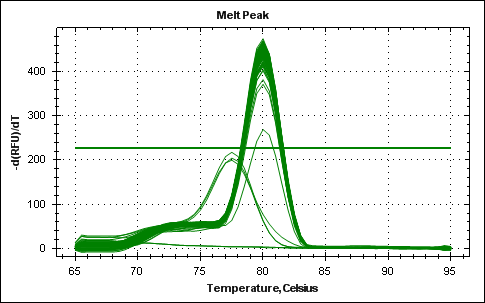


SAND – well-watered SAND – dry

**Results and Data Analysis**

The **delta Cq values** were correlated to the **log2**-**transformed counts** extracted from the data frame of counts in R. Higher counts translate to lower Cq values, indicating an agreement with the trends identified in the WGCNA analysis. Complete Cq values are included as Additional File 16.

**
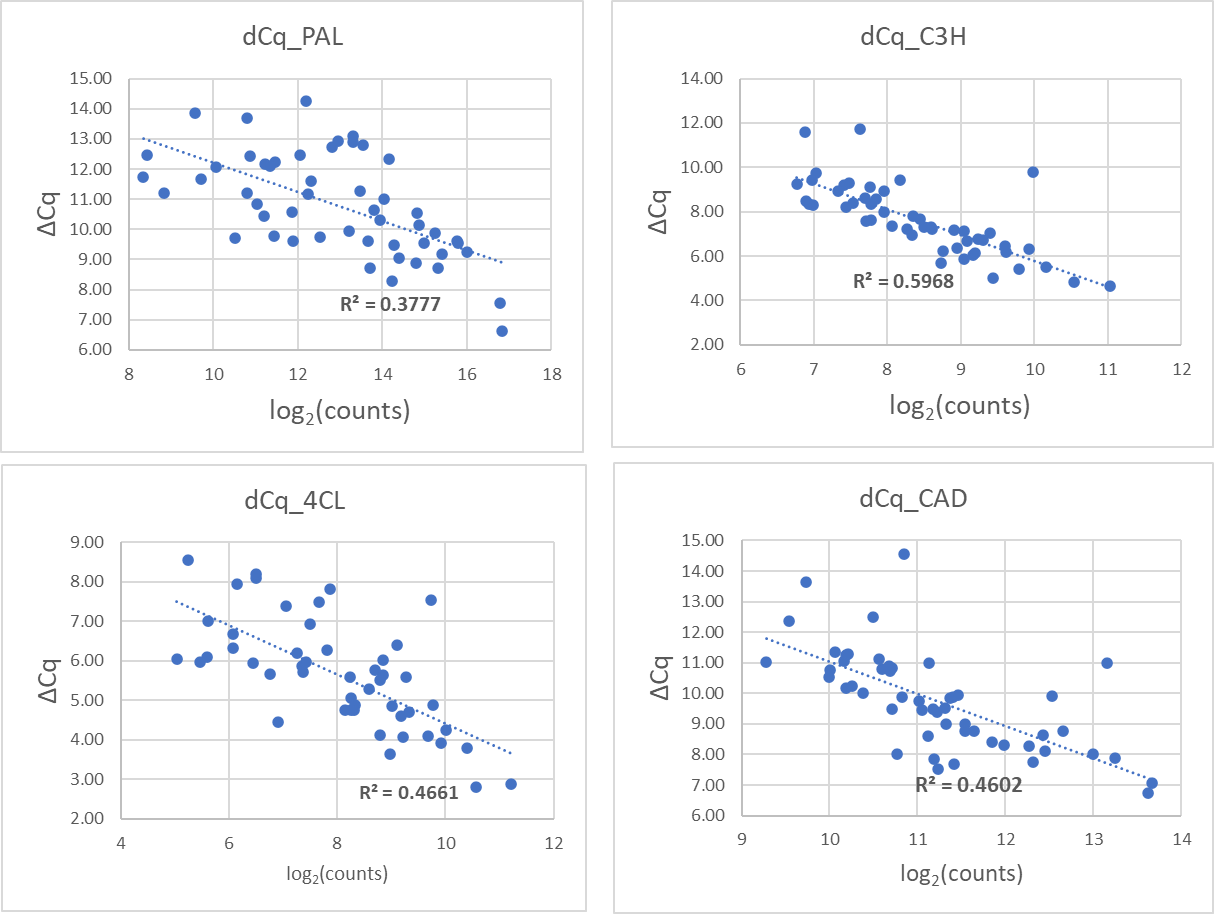
**

Data were analyzed using Microsoft Excel. Outliers were discarded if a Cq could not be detected or if the melt curve indicated a low melting temperature. The geometric mean of two genes, SAND and the 18S rRNA gene, were chosen based on previously published results indicating the stability of their expression both in response to biotic and abiotic conditions [4, 5].

**References**

1. Bustin SA, Benes V, Garson JA, Hellemans J, Huggett J, Kubista M, et al. The MIQE Guidelines: Minimum Information for Publication of Quantitative Real-Time PCR Experiments. *Clin Chem*. 2009;55:611–22.

2. Scully ED, Gries T, Palmer NA, Sarath G, Funnell-Harris DL, Baird L, et al. Overexpression of *SbMyb60* in *Sorghum bicolor* impacts both primary and secondary metabolism. *New Phytol*. 2018;217:82–104.

3. Tetreault HM, Scully ED, Gries T, Palmer NA, Funnell-Harris DL, Baird L, et al. Overexpression of the *Sorghum bicolor SbCCoAOMT* alters cell wall associated hydroxycinnamoyl groups. *PLOS ONE*. 2018;13:e0204153.

4. Zhang K, Niu S, Di D, Shi L, Liu D, Cao X, et al. Selection of reference genes for gene expression studies in virus-infected monocots using quantitative real-time PCR. *J Biotechnol*. 2013;168:7–14.

5. Sudhakar Reddy P, Srinivas Reddy D, Sivasakthi K, Bhatnagar-Mathur P, Vadez V, Sharma KK. Evaluation of Sorghum [Sorghum bicolor (L.)] Reference Genes in Various Tissues and under Abiotic Stress Conditions for Quantitative Real-Time PCR Data Normalization. *Front Plant Sci.* 2016;7. doi:10.3389/fpls.2016.00529.
